# AMPAR dysregulation in microglia drives vascular pathology in diabetic retinopathy via the P2X7R/NLRP3/IL-1β pathway

**DOI:** 10.1186/s12967-026-08195-x

**Published:** 2026-04-27

**Authors:** Lili Zhang, Kaixiang Li, Fengjuan Gao, Jiaojiao Wei, Xin Chen, Gezhi Xu, Yuan Zong, Ting Zhang

**Affiliations:** 1 Eye Institute and Department of Ophthalmology, Eye & ENT Hospital, Fudan University, Shanghai, 200031 China; 2 NHC Key Laboratory of Myopia and Related Eye Diseases, Key Laboratory of Myopia and Related Eye Diseases, Chinese Academy of Medical Sciences, Shanghai, 200031 China; 3 Shanghai Key Laboratory of Visual Impairment and Restoration, Shanghai, 200031 China; 4 Department of Pediatric Cardiothoracic Surgery, Shanghai Children’s Medical Center, Shanghai Jiao Tong University School of Medicine, Shanghai, 200127 China

**Keywords:** Diabetic retinopathy, AMPAR, Calcium-permeable AMPAR, Microglia, P2X7R, NLRP3 inflammasome, IL-1β, Blood-retinal barrier

## Abstract

**Background:**

Diabetic retinopathy (DR) is a leading cause of visual impairment in working-age adults globally, characterized by chronic retinal inflammation and inner blood-retinal barrier (iBRB) disruption. Glutamate excitotoxicity and microglial activation are key pathogenic contributors, but the molecular links from these events to vascular damage remain unclear—particularly the role of α-amino-3-hydroxy-5-methyl-4-isoxazolepropionic acid receptors (AMPARs), whose subunit composition (GluR1–4) regulates calcium permeability.

**Methods:**

Human vitreous humor and retinal tissues, streptozotocin-induced DR mice, high glucose (HG)-stimulated BV2 cells, mouse primary retinal microglia, and bEnd.3 endothelial cells were used. Glutamate levels, AMPAR subunit expression, microglial activation, calcium homeostasis, iBRB integrity and the potential mechanism were assessed via biochemical assays, immunofluorescence, transcriptomics, calcium imaging, Evans blue assays, western blot, and ELISA. Interventions included the AMPAR antagonists (perampanel and NASPM), and an IL-1β neutralizing antibody.

**Results:**

Elevated glutamate levels were observed in the vitreous of DR patients, diabetic mouse retinas, and HG-treated BV2 cells. Critically, a consistent AMPAR subunit composition change (increased GluR1, decreased GluR2) was confirmed in human diabetic retinas, diabetic mouse retinas, HG-treated BV2 cells, and critically, in primary retinal microglia. This subunit change promoted the formation of calcium-permeable AMPARs, triggering downstream events including calcium overload, activation of the ATP/P2X7R/NLRP3 inflammasome pathway, and subsequent upregulation of IL-1β production. The direct link between AMPAR subunit remodeling, elevated intracellular calcium, and increased IL-1β was further substantiated in primary retinal microglia. Ultimately, this cascade impaired iBRB in vivo and enhanced pro-angiogenic responses in endothelial cells in vitro. Notably, both AMPAR inhibition and IL-1β neutralization effectively reversed these pathological changes.

**Conclusions:**

Our findings implicate microglial AMPAR subunit remodeling—favoring Ca²⁺-permeable configuration—as an early trigger of neurovascular inflammation in DR. Targeting the glutamate–AMPAR–P2X7R–IL‑1β cascade may offer a rational strategy to preserve iBRB integrity.

**Graphical Abstract:**

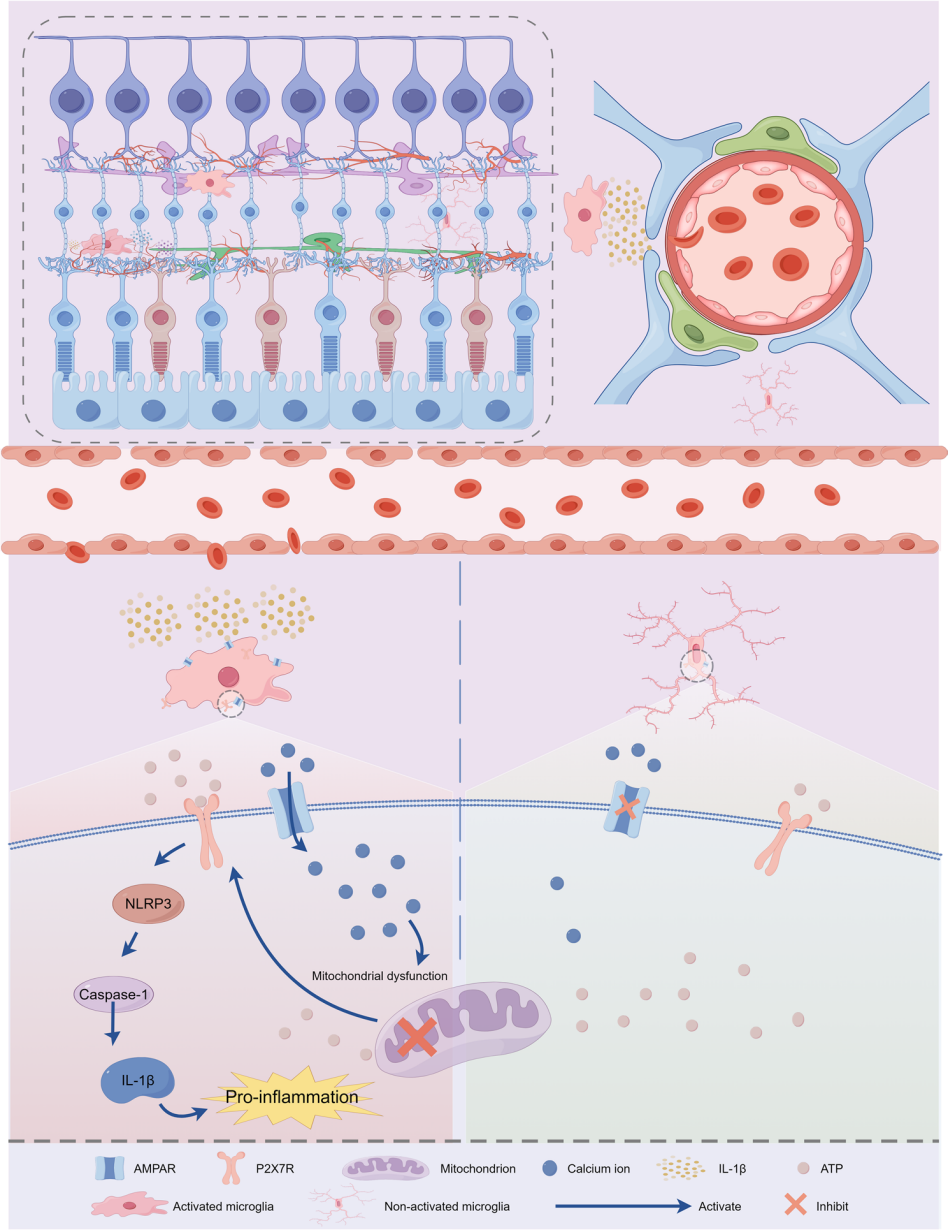

**Supplementary Information:**

The online version contains supplementary material available at 10.1186/s12967-026-08195-x.

## Introduction

Diabetic retinopathy (DR) is a predominant cause of severe visual impairment and blindness among working-age adults globally [1]. Approximately one-third of diabetic patients suffer from DR, which places a heavy burden on patients, their families, and society [2]. Unfortunately, effective treatments targeting the early pathological events of DR to prevent or delay its progression remain scarce.

It has gradually been clarified that DR is not merely a microvascular disease but also characterized by chronic retinal inflammation [3]. A key driver of this inflammatory cascade is the activation of retinal microglia, which occurs early in DR progression and releases large quantities of pro-inflammatory mediators [4], ultimately disrupting the integrity of the inner blood retinal barrier (iBRB)—a hallmark of DR-associated vascular dysfunction [5, 6]. Despite the well-established role of microglial activation in DR pathogenesis, the upstream molecular triggers and downstream signaling cascades mediating this process remain incompletely defined, hindering the development of targeted therapies.

Alongside inflammation, dysregulation of glutamate homeostasis represents another key pathological insult in the diabetic retina [7–10]. Glutamate, the primary excitatory neurotransmitter, is normally cleared by Müller cells [11]. Müller cells take up glutamate via glutamate–aspartate transporter (GLAST) and convert it to glutamine via glutamine synthetase (GS) [12]. Accumulating evidence indicates that retinal glutamate excess in DR animal models predominantly results from hyperglycemia-induced Müller cell dysfunction, characterized by reduced GLAST and GS expression and impaired glutamate cycling, thereby leading to glutamate-induced injury [8–10, 12–14]. Beyond its direct effects on neurons, emerging evidence suggests that glutamate may also signal to microglia, which express functional ionotropic glutamate receptors, including α-amino-3-hydroxy-5-methyl-4-isoxazolepropionic acid receptors (AMPARs) [15, 16].

A critical feature of AMPARs is their variable calcium permeability, which is tightly regulated by subunit composition. Specifically, the presence of the GluR2 subunit renders calcium-impermeable. In pathological states such as ischemia and epilepsy, downregulation of GluR2 expression acts as a ‘molecular switch’, leading to the formation of calcium-permeable AMPARs (CP-AMPARs) and exacerbating glutamate-mediated toxicity [17–19]. Notably, AMPAR dysfunction on microglia has been implicated in the neuroinflammatory processes of various central nervous system (CNS) disorders [20, 21]. However, the specific role of AMPAR signaling in retinal microglia within the context of DR, and how it might bridge the gap between metabolic stress, inflammation, and vascular pathology, remains largely unexplored.

Based on these observations, we hypothesized that diabetes-induced retinal glutamate dysregulation promotes microglial activation via altering AMPAR signaling, which in turn drives endothelial dysfunction and iBRB disruption. Here, we examined this hypothesis and sought to delineate the downstream molecular mechanisms.

## Materials and methods

### Human samples

The human eyes utilized in this study were obtained from the Eye Bank of the Eye and Eye and Ear Nose Throat (ENT) Hospital of Fudan University. The basic information of the patients is listed in Supplementary. Table S1, S2, S3. We complied with all relevant ethical guidelines for working with human participants, and informed consent was obtained from the patients or patients’ family members. The study protocol was approved by the Ethical Oversight Committee of Fudan University Eye and ENT Hospital (approval number: [2023] (2023045)).

### Glutamate measurement

Glutamate levels in human vitreous humor, retinas of DR mice, and BV2 microglial cells were measured using the Glutamate Colorimetric Assay Kit (E-BC-K903-M, Elabscience). Briefly, vitreous humor was collected and centrifuged to obtain supernatants; DR mouse retinas were homogenized in lysis buffer and centrifuged to harvest supernatants; BV2 cells were lysed and centrifuged for clear lysates. After appropriate dilution, 30 µL of supernatant/lysate was added to the reaction mixture following the kit instructions, incubated for 35 min, and the absorbance was detected at 450 nm.

### Experimental animals

Male C57BL/6J mice (8 weeks old) were provided by Vital River Animal Laboratory (Beijing, China). After fasting for 12 h, mice received intraperitoneal administration of 40 mg/kg streptozotocin (STZ; BioFroxx, Munich, Germany) or citrate buffer (vehicle control) for 5 consecutive days. Mice with a blood glucose level exceeding 16.7 mmol/L were considered diabetic. The diabetic mice were randomly given either AMPAR selective antagonist perampanel (PER) (1 mg/kg/week solved in 1% DMSO) or an equivalent dose of 1% DMSO intraperitoneally [22, 23].

### Intravitreal injection

After appropriate anesthesia, pupils were fully dilated with 0.5% tropicamide eye drops. 3 µL aflibercept (40 mg/mL, Eylea), L-glutamate (10 µM), PBS, IL-1β neutralizing antibody or its corresponding isotype control IgG was then administered via intravitreal injection using a 33-gauge microsyringe. The injection was performed 1 mm posterior to the limbus, with the needle inserted at a 30° angle to minimize the risk of retinal damage. Mice were monitored for 30 min post-operation, and a single dose of topical antibiotic eye drops was administered to prevent infection.

### Evans blue assay

After appropriate anesthesia, the mice were administered Evans blue (Sigma, Missouri State, USA) via the femoral vein at a dose of 45 mg/kg. Two hours later, the eyes were enucleated and whole retinal mounts were prepared. The amount and distribution of dye leakage was captured using a fluorescence microscope (Zeiss, Oberkochen, Germany).

### Fundus photography

Following appropriate anesthesia, pupils were fully dilated, and the ocular surface was kept moist with sterile artificial tears to prevent corneal dryness. Mice were placed in a prone position on a custom platform, and their heads were gently stabilized with an adjustable holder to minimize movement artifacts. Fundus images were acquired using the OPTO-RIS system (Optoprobe, England) at a fixed magnification under standardized illumination conditions, with a focus on the retinal posterior pole.

### RNA sequencing and bioinformatics analysis

Retinal tissues were collected from DR mice and DR mice treated with PER (*n* = 5 per group). Total RNA was extracted and 1 µg of high-quality RNA was used for library construction with the Illumina TruSeq™ RNA Sample Preparation Kit. Raw paired-end reads were processed by trimming adapters (SeqPrep), quality-controlling with Sickle, aligning to the mouse reference genome, assembling into transcripts (StringTie), and quantifying as transcripts per million reads (TPM). Differentially expressed genes between DR and DR + PER were identified using DESeq2 (version 3.11) with |log2 fold change| ≥ 1 and adjusted *P* < 0.05. Gene Ontology (GO) enrichment analysis was performed via the clusterProfiler R package (biological process, cellular component, molecular function categories; adjusted *P* < 0.05), and Gene Set Enrichment Analysis (GSEA) was conducted with WikiPathways gene sets, with pre-defined gene sets based on prior biological knowledge.

### Cell culture

The BV2 cells and the bEnd.3 cells were obtained from the Department of Ophthalmology and Vision Science, Eye and ENT Hospital, Fudan University. Both cells were cultured in Dulbecco’s modified Eagle’s medium/F12 (Procell, Wuhan, China) with 10% fetal bovine serum (FBS) (Gibco, California, USA).

Primary retinal microglia were isolated from the eyes of 5‑day‑old C57BL/6J mice. Retinas were dissected and washed with pre‑warmed DMEM/F12 medium. Following 10 min trypsin digestion, the reaction was stopped by adding complete culture medium (DMEM/F12 supplemented with 15% FBS and 1% penicillin‑streptomycin). The cell suspension was centrifuged at 1000 rpm for 5 min, and the cell pellet was resuspended and seeded into T75 flasks. After 14 days of culture, Müller cells were spread all over the bottom of the plate, while retinal microglia appeared as small, round, bright cells loosely adhering to the Müller cell layer [24]. Flasks were shaken on an orbital shaker at 37 °C for 1 h. The supernatant was collected and centrifuged at 1000 rpm for 5 min. Isolated microglia were then seeded into 24‑well plates for subsequent experiments.

BV2 cells and primary retinal microglia were exposed to one of the following conditions for 48 h: low glucose (LG, 5 mM D-glucose), high glucose (HG, 30 mM D-glucose), and high mannitol (HM, 5 mM D-glucose plus 25 mM mannitol; an osmotic control). The AMPAR inhibitor PER and CP‑AMPAR inhibitor NASPM (1-naphthylacetyl spermine trihydrochloride) were administered at 10 µM and 25 µM, respectively [23, 25].

Conditioned media (CM) were then collected from BV2 cells under LG, HG, or HG + PER conditions. After centrifugation, the CM was used to culture bEnd.3 cells for 24 h. For the HG-CM group, parallel treatments were performed by adding either an isotype control IgG or an IL-1β neutralizing antibody (1 µg/mL) to the CM. Accordingly, the five types of CM are designated as LG-CM, HG-CM, HG-PER-CM, HG-CM-IgG, and HG-CM-anti-IL-1β.

### Immunofluorescence

For the preparation of frozen sections, the fixed eyes were embedded in optimal cutting temperature compound (Sakura, California, USA) after dehydration. Immunofluorescence (IF) was performed on 5-µm tissue sections. Then, for different purposes, the frozen sections, retinas and cells were fixed in 4% paraformaldehyde and blocked for 1 h in blocking buffer (5% BSA and 1% Triton X-100 dissolved in PBS), followed by sequential incubation with primary antibodies and appropriate fluorescently labeled secondary antibodies. All the antibodies used in this study were shown in Supplementary. Table S4. Images were acquired using a fluorescence microscope (Zeiss) and a confocal microscope (Leica, Wetzlar, Germany).

### Proliferation assay

The proliferative ability of the bEnd.3 cells was determined using an EDU detection kit (Beyotime, Shanghai, China) following the manufacturer’s instructions. Images were captured using a fluorescence microscope (Zeiss).

### Tube formation assay

BEnd.3 cells were seeded into the Matrigel-coated (Corning, New York, USA) 24-well plates at a density of 5 × 10^5^ cells/well. After incubation, images were captured with a microscope (Zeiss).

### Wound healing assay

After bEnd.3 cells had grown to confluence, scratches were made and serum-free medium was replaced. Images of the wounds were taken immediately and 24 h after the scratching using a microscope (Zeiss).

### Transwell assay

Cells were suspended in 100 µL serum-free medium and added to the upper chamber of the Transwell inserts ((8-µm pore size) (BD Falcon, New Jersey, USA). The lower chamber was filled with medium containing 10% FBS. After incubation, the non-migrated cells were removed and the migrated cells were stained with 0.1% crystal violet. The migrating cells were imaged using an inverted microscope (Zeiss).

### Real-time PCR

Total RNA was extracted from BV2 cells using a FastPure Cell/Tissue Total RNA Isolation kit (Vazyme, Nanjing, China). The extracted RNA was reverse transcribed by HiScript Q RT SuperMix for qPCR kit (Vazyme). qRT-PCR was performed with ChamQ SYBR qPCR Master Mix (Vazyme). β-actin was used as an internal control. Quantification was performed using the 2^−ΔΔCt^ method. The primer sequences are listed in Supplementary. Table S5.

### Intracellular calcium imaging

BV2 cells and primary retinal microglia were loaded with the calcium indicator Fluo-4 AM (Beyotime) following the manufacturer’s instructions. 10 mM BAPTA-AM (1,2-Bis(2-aminophenoxy) ethane-N, N,N, N-tetraacetic acid tetrakis(acetoxymethyl ester)) was used as a positive control. Flow cytometry (BD, Franklin Lakes, NJ, USA) and confocal microscopy (Leica) were employed to quantify the Fluo4-AM as an index of intracellular Ca^2+^ levels.

### Mitochondrial membrane potential

The mitochondrial membrane potential of the BV2 cells was determined using a JC-1 assay kit (Beyotime) in accordance with the manufacturer’s protocol. Signals were visualized with a confocal microscope (Leica).

### Measurement of ATP

The extracellular and intracellular ATP levels of BV2 cells were measured using an ATP bioluminescence assay kit (Beyotime) in accordance with the manufacturer’s instructions.

### Western blotting

Proteins were separated by electrophoresis on sodium dodecyl sulfate–polyacrylamide gels and then transferred onto polyvinylidene fluoride membranes. After blocking with 5% BSA, the membranes were incubated overnight with primary antibodies, followed by the corresponding secondary antibody. Bands were detected using a chemiluminescence reagent (Millipore, Massachusetts, USA) under the ChemiDoc MP Imaging System (Bio-Rad, California, USA).

### ELISA assay

Secretory protein levels of IL-1β in BV2 cells and primary retinal microglia were measured using an ELISA kit (Servicebio, Wuhan, China), following the manufacturer’s instructions.

### Statistical analysis

Data were analyzed as detailed in figure legends and as appropriate for each experiment by using unpaired Student’s *t*-test, unpaired *t*-test with Welch’s correction, one-way analysis of variance (ANOVA) with Tukey’s post-hoc tests, Welch’s ANOVA with Dunnett’s T3 test, and Kruskal–Wallis test with Dunn’s test. Data were presented as mean ± standard deviation (SD). *P* < 0.05 was accepted as statistically significant.

## Results

### Glutamate elevation and AMPAR subunit composition change in human diabetic retinopathy

**Fig. 1:**
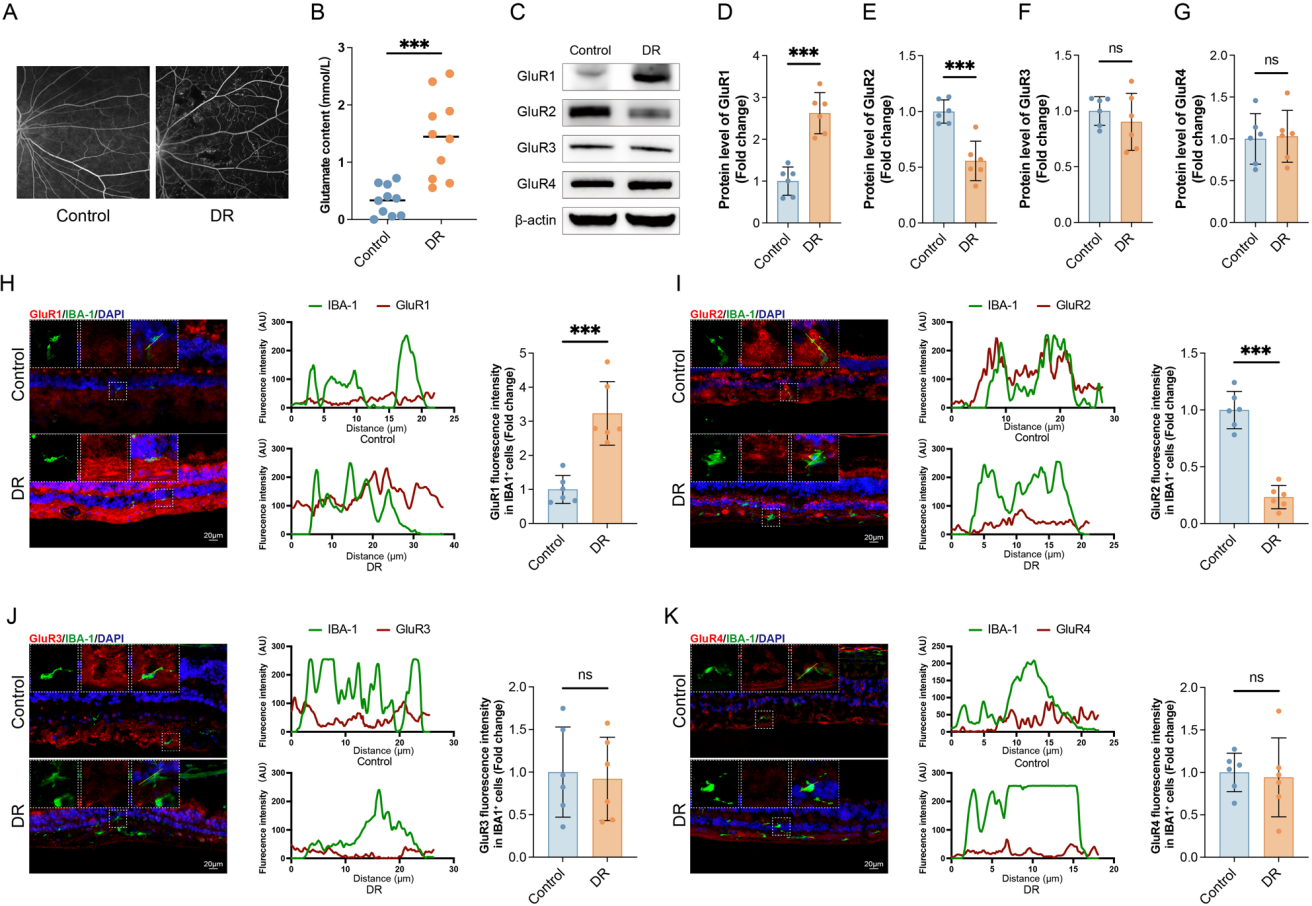
Glutamate elevation and AMPAR subunit composition change in human diabetic retinopathy.(**A**) Representative fundus fluorescein angiography of DR patients, showing non-perfusion areas and vascular leakage. (**B**) Glutamate levels in the vitreous humor of DR patients and control patients with epiretinal membrane. *n *= 10. ***P < 0.001. Unpaired *t*-test with Welch’s correction. (**C**–**G**) Representative western blot images and quantitative analysis of AMPAR subunits (GluR1–4) in human retinas. *n* = 6. ***P < 0.001; ns, not significant. Unpaired Student *t*-test. (H–K) Representative immunofluorescence staining of AMPAR subunits in frozen sections of human retinas, with quantitative analysis of the fluorescence intensity of GluR1–4 in IBA-1^+^ cells. Scale bar: 20 μm. *n* = 6. ***P < 0.001; ns, not significant. Unpaired Student* t*-test. DR: diabetic retinopathy

To investigate the role of glutamate-AMPAR signaling in DR, we first analyzed human clinical samples. Fundus fluorescein angiography of DR patients revealed characteristic microvascular lesions, including non-perfusion areas and vascular leakage (Fig. 1A). Glutamate levels were significantly increased in the vitreous humor of DR patients compared to control patients with epiretinal membrane (Fig. 1B).​ Western blot analysis showed significantly increased GluR1 and decreased GluR2 expression in the retinas of DR patients compared with controls. GluR3 and GluR4 levels did not differ significantly between the two groups (Fig. 1C–G). IF staining of human retinal frozen sections was performed to evaluate the expression and localization of AMPAR subunits (Fig. 1H–K). Key observations included: GluR1 and GluR2 both colocalized with IBA-1⁺ microglia in DR retinas, with quantitative analysis confirming significantly increased GluR1 fluorescence intensity and markedly decreased GluR2 fluorescence intensity in IBA-1⁺ cells in DR. GluR3 and GluR4 fluorescence intensities in IBA-1⁺ cells showed no significant alterations. These data indicate microglial CP-AMPAR formation in the diabetic retina.

### Dysregulated AMPAR signaling mediates hyperglycemia-induced microglial activation in vivo

**Fig. 2:**
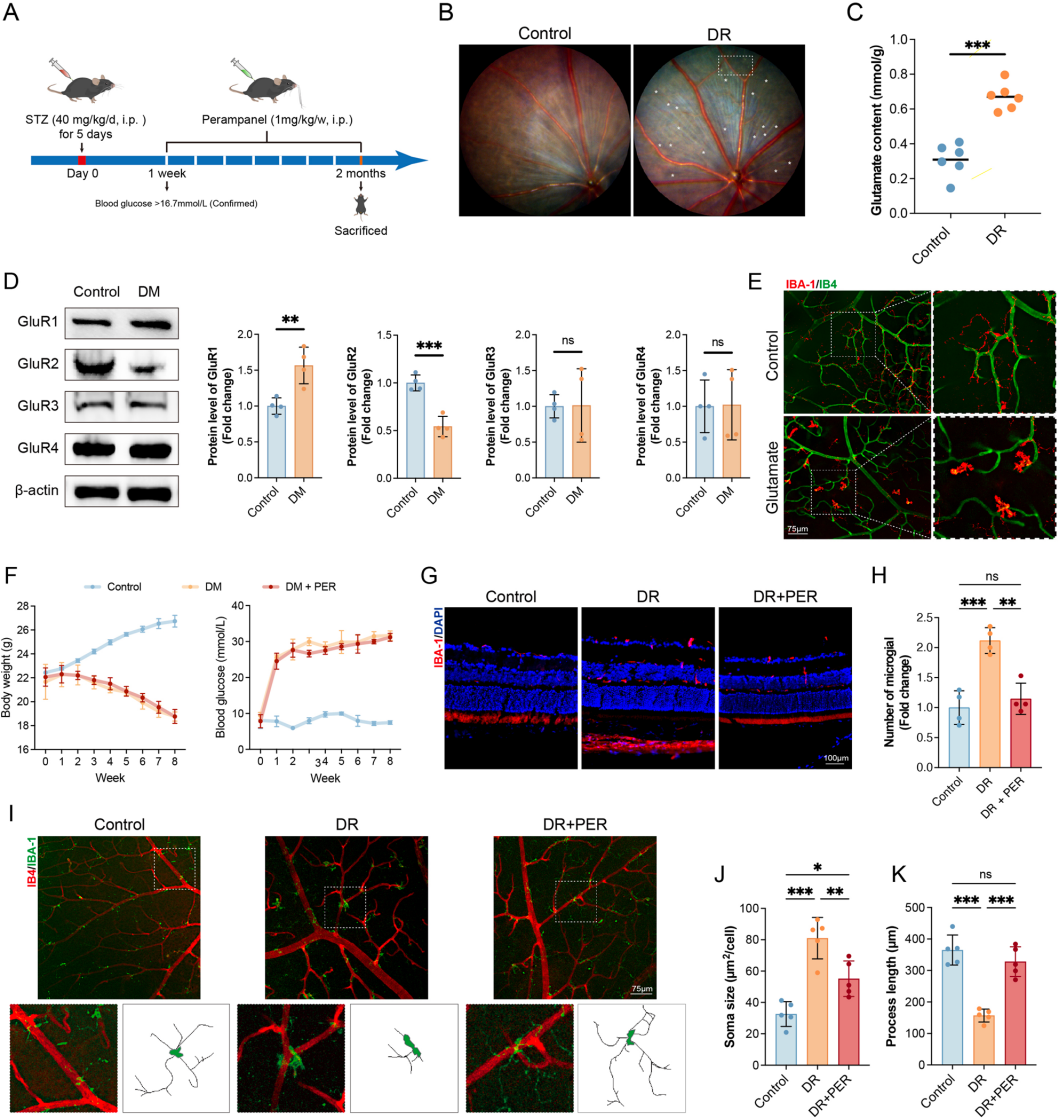
Dysregulated AMPAR signaling mediates hyperglycemia-induced microglial activation in vivo. (**A**) Schematic of DR model establishment in mice. (**B**) Fundus photography of DR mice and age-matched littermates; dashed box indicates hard exudates and asterisks mark small hemorrhages. (**C**) Retinal glutamate levels in DR mice and age-matched controls.* n* = 6. ***P < 0.001. Unpaired Student *t*-test. (**D**) Representative western blot images and quantitative analysis of AMPAR subunits (GluR1–4) in retinas of DR mice and controls. *n *= 4. **P < 0.01; ***P < 0.001; ns, not significant. Unpaired Student *t*-test. (**E**) Immunofluorescence staining of IBA-1 (microglia) and IB4 (vascular marker) in retinas of glutamate-injected and PBS-injected mice. Scale bar: 75 μm. (**F**) Fluctuations in blood glucose and body weight among control mice, DR mice, and DR mice treated with PER. (**G**–**H**) Immunofluorescence staining for IBA-1 in frozen retinal sections and quantitative analysis of IBA-1‑positive cell numbers. Scale bar: 100 μm. *n* = 4. **P < 0.01; ***P < 0.001; ns, not significant. One-way ANOVA with Tukey’s test. (I–K) Immunofluorescence staining of IBA-1 and IB4 in retinas of controls, DR mice, and DR + PER mice, with quantitative analysis of microglial process length and soma size. Scale bar: 75 μm. *n *= 5. *P < 0.05; **P < 0.01; ***P < 0.001; ns, not significant. One-way ANOVA with Tukey’s test. DR: diabetic retinopathy; PER: perampanel

To validate the human findings, DR mouse models were established by intraperitoneal injection of STZ (Fig. 2A). Fundus photography showed typical DR microangiopathy in diabetic mice, including hard exudates and small hemorrhages (Fig. 2B). In the STZ-induced DR model, retinal glutamate levels were elevated (Fig. 2C). Western blot analysis confirmed the composition change of AMPAR subunits, with significantly increased GluR1 and decreased GluR2 protein levels (Fig. 2D). Direct validation of glutamate’s effect on microglia showed that intravitreal injection of glutamate in healthy mice induced an amoeboid (activated) microglial morphology, whereas PBS-injected mice exhibited ramified (resting) microglia (Fig. 2E).

To determine the functional contribution of AMPAR signaling to this phenotype, we administered the AMPAR antagonist PER. Pharmacological inhibition of AMPAR activity didn’t affect systemic glycemia or weight of the DR mice (Fig. 2F). IF staining of retinal frozen sections demonstrated an increased number of IBA-1⁺ microglia in diabetic mice, which was significantly reduced by PER (Fig. 2G–H). Retinal flat-mount IF staining further revealed that diabetic mice had IBA-1⁺ microglia with increased soma area and decreased process length, and these morphological abnormalities were effectively reversed by PER (Fig. 2I–K).

### High glucose drives a pro-inflammatory microglial phenotype through AMPAR subunit dysregulation in vitro

**Fig. 3:**
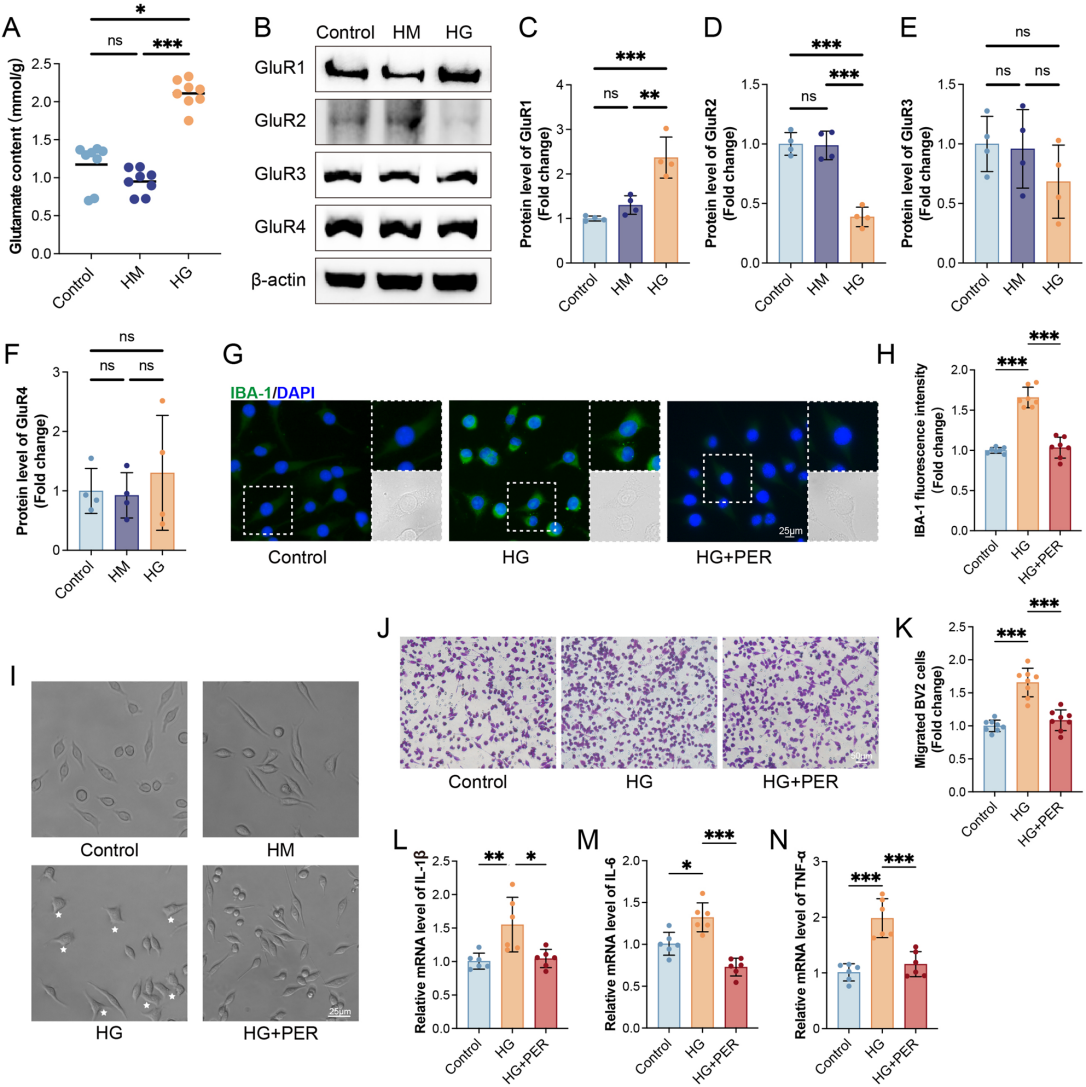
High glucose drives a pro-inflammatory microglial phenotype through AMPAR subunit dysregulation in vitro. (**A**) Glutamate levels in BV2 microglial cells under different treatments.* n* = 8. *P < 0.05; ***P < 0.001; ns, not significant. Kruskal–Wallis test with Dunn's test. (**B**–**F**) Representative western blot images and quantitative analysis of AMPAR subunits (GluR1–4) in BV2 cells. *n* = 4. **P < 0.01; ***P < 0.001; ns, not significant. One-way ANOVA with Tukey's test. (**G**) Representative immunofluorescence staining of IBA-1 and corresponding brightfield images in BV2 cells. Scale bar: 25 μm. (**H**) Quantitative analysis of IBA-1 staining intensity in BV2 cells. *n* = 7. ***P < 0.001. One-way ANOVA with Tukey's test. (**I**) Microscopic images of BV2 cells; asterisks indicate the ameboid-like BV2 cells. Scale bar: 25 μm. (**J**–**K**) Transwell assay showing the migratory capacity of BV2 cells with different treatments, with quantitative analysis. Scale bar: 50 μm. *n* = 8. ***P < 0.001. One-way ANOVA with Tukey's test. (**L**) Relative mRNA expression levels of IL-1β in BV2 cells. *n* = 6. *P < 0.05; **P < 0.01. One-way ANOVA with Tukey's test. (**M**) Relative mRNA expression levels of IL-6 in BV2 cells. *n* = 6. *P < 0.05; ***P < 0.001. Welch's ANOVA with Dunnett's T3 test. (**N**) Relative mRNA expression levels of TNF-α in BV2 cells. *n* = 6. ***P < 0.001. One-way ANOVA with Tukey's test. HM: high mannitol (osmotic control); HG: high glucose; PER: perampanel

HG stimulation significantly elevated the glutamate level in BV2 microglial cells (Fig. 3A). Western blot assays showed that HG-stimulated BV2 cells recapitulated the in vivo AMPAR expression pattern: GluR1 was significantly upregulated, GluR2 was markedly downregulated, while GluR3 and GluR4 showed no significant changes (Fig. 3B–F). To better approximate the phenotype of retinal microglia, we isolated primary mouse retinal microglia (Supplementary Fig. S1A–B). Western blot results were consistent with those in BV2 cells: HG induced significant upregulation of GluR1 and marked downregulation of GluR2, with no significant changes in GluR3 or GluR4 (Supplementary Fig. S1C–G).

Subsequent functional assays revealed that HG increased IBA-1 fluorescence intensity in BV2 cells and induced an amoeboid morphology, whereas control and hyperosmolar groups maintained a spindle-shaped resting state; targeting AMPAR signaling with PER reduced the HG-elevated IBA-1 fluorescence intensity and reversed the HG-induced morphological change (Fig. 3G–I). Transwell assays demonstrated enhanced migration of HG-stimulated BV2 cells, which was suppressed by PER (Fig. 3J–K). qPCR results showed that HG upregulated mRNA levels of inflammatory cytokines (IL-1β, IL-6, TNF-α) in BV2 cells, and these effects were abrogated by PER (Fig. 3L–N). Importantly, hyperosmolar conditions did not significantly affect BV2 cell migration, IBA-1 expression, or inflammatory cytokine levels, excluding osmotic stress as a confounding factor (Supplementary Fig. S2).

### AMPAR/calcium-driven mitochondrial dysfunction in metabolically stressed microglia

**Fig. 4:**
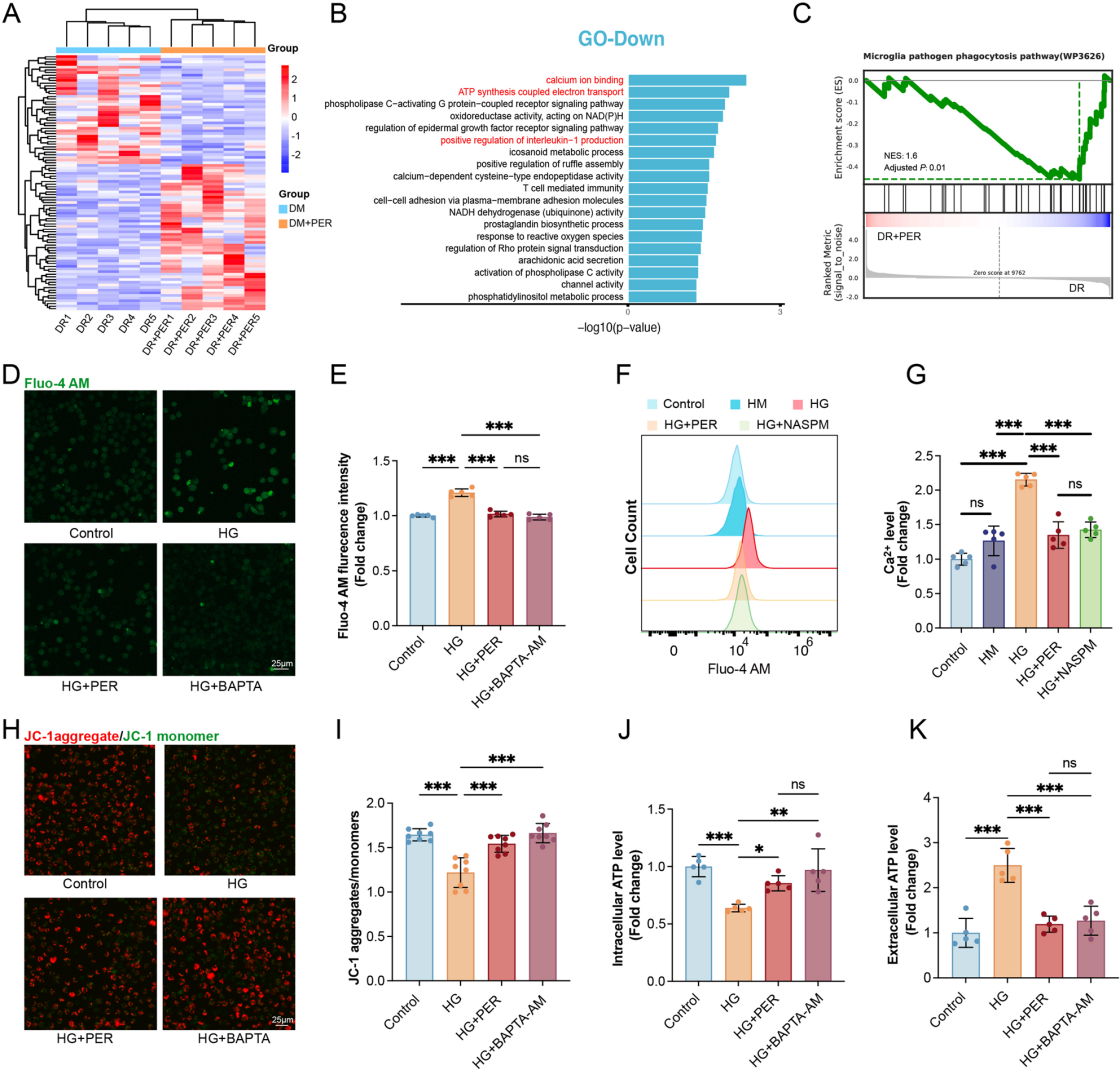
AMPAR/calcium-driven mitochondrial dysfunction in metabolically stressed microglia. (**A**) Heat map showing hierarchical clustering of differentially expressed genes identified by RNA sequencing in retinas of DR and DR + PER mice. *n *= 5. (**B**) Gene Ontology (GO) enrichment analysis of downregulated differentially expressed genes in DR + PER mice. (**C**) Gene Set Enrichment Analysis identifying the “microglia pathogen phagocytosis pathway”. (**D**–**E**) Fluo-4 AM fluorescence imaging detecting intracellular calcium levels in BV2 cells. Scale bar: 25 μm. *n *= 5. ***P < 0.001; ns, not significant. One-way ANOVA with Tukey’s test. (**F**–**G**) Fluo-4 AM flow cytometry analysis of intracellular calcium levels in BV2 cells. *n *= 5. ***P < 0.001; ns, not significant. One-way ANOVA with Tukey’s test. (**H**–**I**) JC-1 staining of BV2 cells (assessing mitochondrial membrane potential) and quantitative analysis. Scale bar: 25 μm. *n* = 8. ***P < 0.001. One-way ANOVA with Tukey’s test. (**J**–**K**) Measurement of intracellular and extracellular ATP concentrations of BV2 cells. *n* = 5. *P < 0.05; **P < 0.01; ***P < 0.001; ns, not significant. One-way ANOVA with Tukey’s test. DR: diabetic retinopathy; PER: perampanel; HM: high mannitol; HG: high glucose; NASPM: 1-naphthylacetyl spermine trihydrochloride; BAPTA-AM: 1,2-Bis(2-aminophenoxy) ethane-N, N,N, N-tetraacetic acid tetrakis(acetoxymethyl ester)

Transcriptomic analysis revealed that, compared with the DR group, inhibiting AMPAR activity downregulated genes related to “calcium ion binding”, “ATP synthesis coupled electron transport”, and “positive regulation of IL-1 production” (Fig. 4A–B). GSEA based on the WikiPathways database was conducted to investigate biological pathway alterations induced by AMPAR inhibition, which identified notable enrichment of the “microglia pathogen phagocytosis pathway” (Fig. 4C). While generated from whole-retina samples and thus lacking cell-type resolution, these findings led us to explore the mechanisms responsible for microglial dysfunction under diabetic conditions, as well as the microglial response to AMPAR inhibition.

Accordingly, based on the observed shift toward GluR2‑lacking AMPARs and the above transcriptomic findings, we assessed intracellular calcium levels and mitochondrial function in cultured microglial cells. Fluo-4 AM calcium imaging and flow cytometry revealed abnormal intracellular calcium influx in HG-stimulated BV2 cells, which was abrogated by PER or BAPTA-AM (Fig. 4D–E; Supplementary Fig. S3A–B). Furthermore, NASPM also effectively attenuated HG-induced Ca²⁺ influx in BV2 cells and primary retinal microglia (Fig. 4F–G; Supplementary Fig. S1H–J). JC-1 staining showed that HG decreased the JC-1 aggregate/monomer ratio, indicating mitochondrial depolarization, and this dysfunction was reversed by PER or BAPTA-AM (Fig. 4H–I). Additionally, HG caused reduced intracellular ATP levels and increased extracellular ATP release in BV2 cells, both of which were restored by PER or BAPTA-AM (Fig. 4J–K). Collectively, these findings demonstrate that AMPAR inhibition mitigates mitochondrial dysfunction in HG-stimulated microglia via suppressing intracellular calcium influx.

### Calcium-dependent activation of the P2X7R-NLRP3 inflammasome pathway in high glucose-treated microglia

**Fig. 5:**
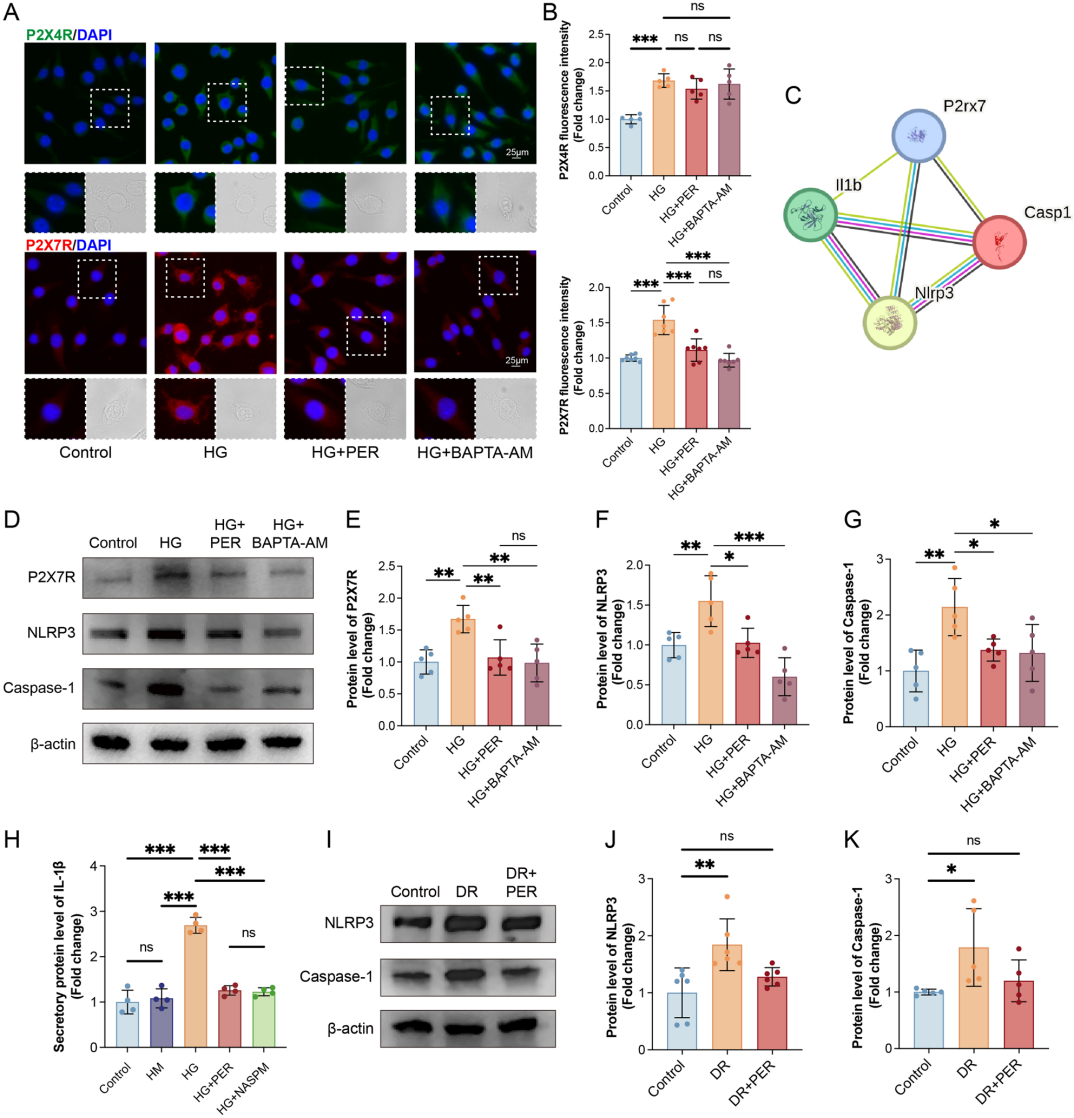
Calcium-dependent activation of the P2X7R-NLRP3 inflammasome pathway in high glucose-treated microglia. (**A**) Representative immunofluorescence staining of P2X4R/P2X7R and corresponding brightfield images in BV2 cells. Scale bar: 25 μm. (**B**) Quantitative analysis of P2X4R and P2X7R staining intensity in BV2 cells.* n* = 5–7. ***P < 0.001; ns, not significant. One-way ANOVA with Tukey’s test. (**C**) Protein-protein interaction network of P2X7R analyzed using STRING (string-db.org). (**D**–**G**) Representative western blot images and quantitative analysis of P2X7R, NLRP3, and Caspase-1 protein expression in BV2 cells. *n *= 5. *P < 0.05; **P < 0.01; ***P < 0.001; ns, not significant. One-way ANOVA with Tukey’s test. (**H**) ELISA-based quantification of secreted IL-1β levels in BV2 cell supernatants. *n *= 4. ***P < 0.001; ns, not significant. One-way ANOVA with Tukey’s test. (**I**) Representative western blot images of NLRP3 and Caspase-1 protein expression in retinas of control mice, DR mice, and DR mice treated with PER. (**J**) Quantitative analysis of NLRP3 protein expression. *n* = 6. **P < 0.01; ns, not significant. Kruskal–Wallis test with Dunn’s test. (**K**) Quantitative analysis of Caspase-1 protein expression. *n* = 5. *P < 0.05; ns, not significant. One-way ANOVA with Tukey’s test. HM: high mannitol; HG: high glucose; PER: perampanel; DR: diabetic retinopathy; NASPM: 1-naphthylacetyl spermine trihydrochloride; BAPTA-AM: 1,2-Bis(2-aminophenoxy) ethane-N, N,N, N-tetraacetic acid tetrakis(acetoxymethyl ester)

Extracellular ATP functions as a pro-inflammatory signal by activating purinergic receptors, and both P2X4R and P2X7R are implicated in retinal inflammation [26–28]. IF staining showed that HG upregulated P2X4R and P2X7R expression in BV2 cells. Notably, only the upregulation of P2X7R was reversed by PER or BAPTA-AM (Fig. 5A–B, D–E). This pointed to a specific, AMPAR/calcium-dependent regulation of P2X7R. Given that P2X7R is a potent activator of the NLRP3 inflammasome—a key driver of caspase-1 activation and IL-1β maturation [29], we investigated this downstream axis. STRING protein interaction analysis supported a close network association between P2X7R and NLRP3 inflammasome components (Fig. 5C). Consistent with this link, HG stimulation significantly increased the protein levels of NLRP3, caspase-1 in BV2 cells. Crucially, these increases were attenuated by either PER or BAPTA-AM (Fig. 5D–G). In line with these results, ELISA further demonstrated that HG‑induced IL‑1β secretion was effectively suppressed by PER or NASPM, in both BV2 cells and primary retinal microglia (Fig. 5H; Supplementary Fig. S1K; Supplementary Fig. S3C).

This NLRP3 signaling pathway was also relevant in vivo. The protein levels of NLRP3 and caspase-1 were elevated in the retinas of DR mice, and this upregulation was suppressed by PER treatment (Fig. 5I–K). To clarify the cellular source of IL-1β in retinal tissues, IF co-staining of human retinal sections revealed that IL-1β was predominantly co-localized with the microglial marker IBA-1 in DR (Supplementary Fig. S4), confirming microglia as an important source of IL-1β in diabetic retinas. Collectively, these data indicate that AMPAR-mediated calcium influx promotes microglial inflammation by specifically upregulating the P2X7R/NLRP3/caspase-1 pathway, leading to increased IL-1β production.

### Microglia-derived IL-1β mediates endothelial angiogenic behavior in a paracrine manner

**Fig. 6:**
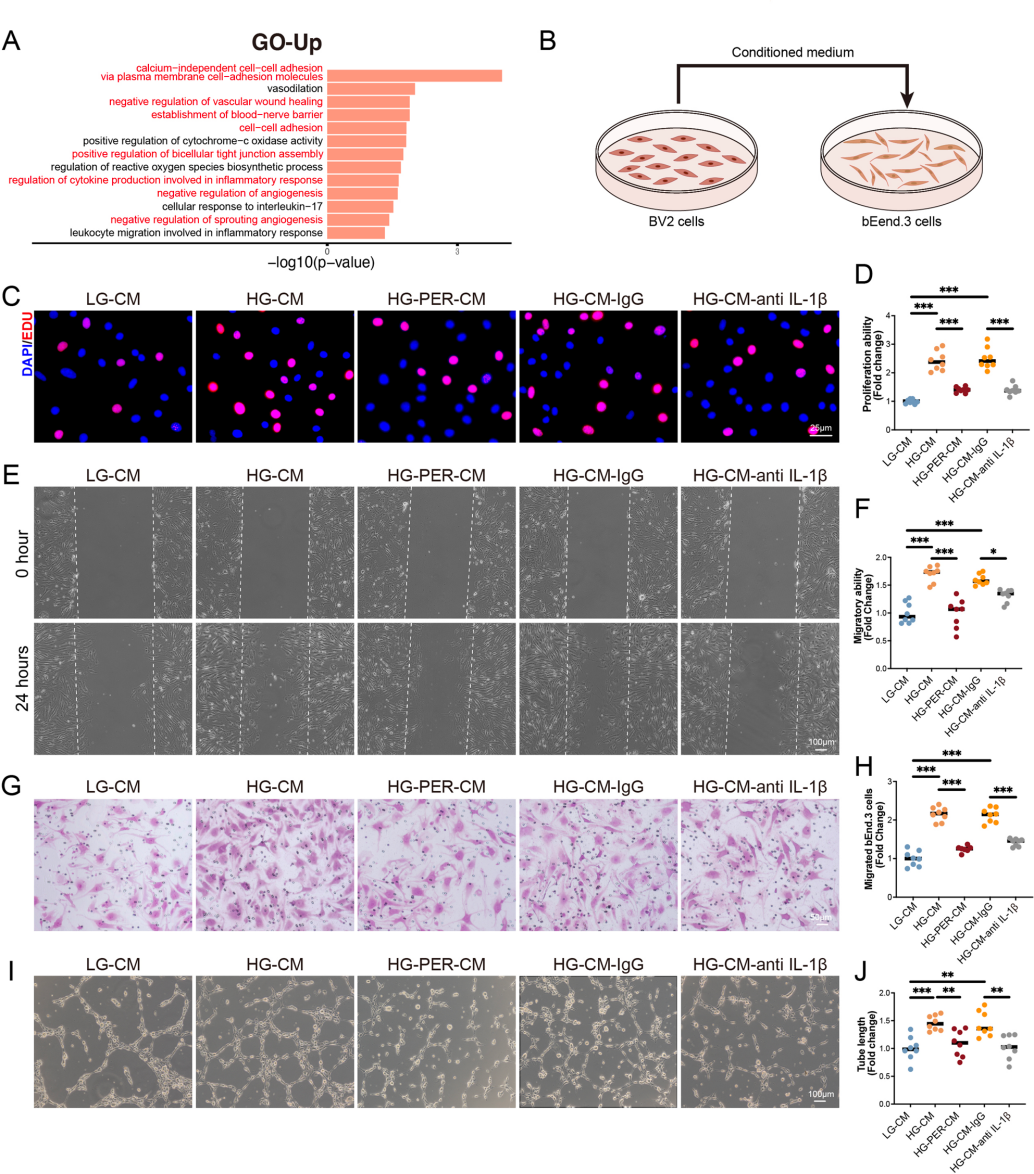
Microglia-derived IL-1β mediates endothelial angiogenic behavior in a paracrine manner. (**A**) Gene Ontology (GO) enrichment analysis of upregulated differentially expressed genes in DR + PER mice. (**B**) Schematic of the co-culture system: bEnd.3 endothelial cells were cultured with conditioned medium from BV2 microglial cells. (**C**–**D**) EDU assay evaluating the proliferative capacity of bEnd.3 cells. Scale bar: 25 μm.* n* = 8. ***P < 0.001. Welch’s ANOVA with Dunnett’s T3 test. (**E**–**F**) Wound healing assay assessing the migratory capacity of bEnd.3 cells. Scale bar: 100 μm. *n* = 8. *P < 0.05; ***P < 0.001. One-way ANOVA with Tukey’s test. (**G**–**H**) Transwell assay detecting the migratory capacity of bEnd.3 cells. Scale bar: 50 μm.* n* = 8. ***P < 0.001. One-way ANOVA with Tukey’s test. (**I**–**J**) Tube formation assay evaluating the angiogenic capacity of bEnd.3 cells. Scale bar: 100 μm.* n *= 8. **P < 0.01; ***P < 0.001. One-way ANOVA with Tukey’s test. LG: low glucose; HG: high glucose; CM: conditioned medium

Upregulated genes in PER-treated DR mice were associated with negative regulation of angiogenesis and endothelial tight junctions (Fig. 6A). We then investigated whether activated microglia could exert a paracrine effect on endothelial cells. To this end, we established a BV2-bEnd.3 indirect co-culture system, where bEnd.3 endothelial cells were treated with CM from BV2 cells (Fig. 6B).

Functional assays demonstrated that, compared with LG-CM, HG-CM significantly promoted bEnd.3 cell proliferation, migration, and tube formation—key hallmarks of pro-angiogenic activity in endothelial cells (Fig. 6C–J). Notably, these HG-induced pro-angiogenic responses were completely abrogated when bEnd.3 cells were treated with HG-PER-CM or when IL-1β was neutralized in the HG-CM (Fig. 6C–J). Direct treatment of bEnd.3 cells with PER alone did not affect their proliferative, migratory, or tube-forming abilities (Supplementary Fig. S5), ruling out a direct regulatory effect of PER on endothelial cells. This confirms that the observed endothelial protection is mediated indirectly through the suppression of microglial activation and subsequent IL-1β release.

### AMPAR/IL-1β blockade alleviates blood-retinal barrier disruption in DR

**Fig. 7:**
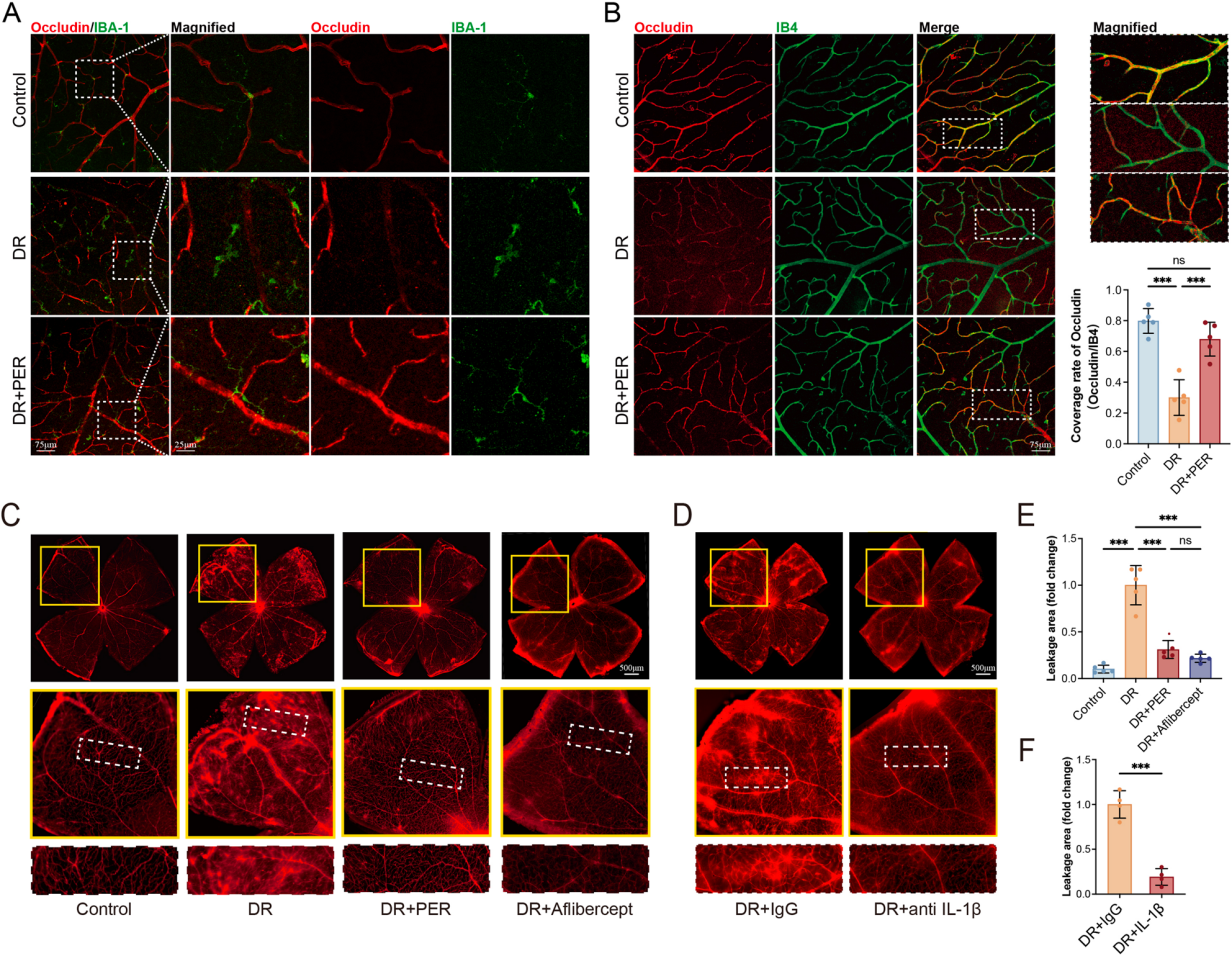
AMPAR/IL-1β blockade alleviates blood-retinal barrier disruption in DR. (**A**) Immunofluorescence staining of flat-mounted retinas with antibodies against IBA-1 (microglia) and Occludin (tight junction protein). Scale bar: 75 μm; magnified scale bar: 25 μm. (**B**) Representative immunofluorescence images and quantitative analysis of Occludin coverage in retinal blood vessels (calculated as Occludin^+^/IB4^+^ ratio) among controls, DR mice, and DR + PER mice. Scale bar: 75 μm. *n* = 5. ***P < 0.001; ns, not significant. One-way ANOVA with Tukey’s test. (**C**–**F**) Evans blue assay: representative fluorescence images and quantitative analysis of retinal vascular leakage. Scale bar: 500 μm. *n* = 4–5. E: ***P < 0.001; ns, not significant. One-way ANOVA with Tukey’s test. F: ***P < 0.001. Unpaired Student *t*-test. DR: diabetic retinopathy; PER: perampanel

Spatial analysis revealed that activated microglia colocalized with sites of Occludin loss in diabetic retinas (Fig. 7A). Temporally, activated microglia were observed in the retinas of diabetic mice at 4 weeks—earlier than the occurrence of iBRB disruption—indicating that this microglial activation precedes detectable breakdown of the iBRB (Supplementary Figs. S6, S7).

To dissect the functional consequences of microglial activation and glutamate-AMPAR signaling dysregulation on iBRB integrity, we evaluated retinal tight junction protein expression and vascular permeability in DR mice.​ Quantitative analysis of Occludin vascular coverage confirmed that the ratio of Occludin⁺ area to IB4⁺ vascular area was significantly reduced in DR compared to controls, which was restored by PER (Fig. 7B). Functional assessment using the Evans blue assay corroborated these structural findings, demonstrating a marked increase in vascular leakage in DR that was significantly attenuated by both AMPAR blockade and aflibercept (as a positive control) (Fig. 7C, E). Critically, the protective effect on barrier function was replicated by intravitreal injection of an IL-1β neutralizing antibody (Fig. 7D, F). This result, together with the observed colocalization and temporal precedence, positions IL-1β—released via the AMPAR/P2X7R/NLRP3 pathway in activated microglia—as a key paracrine mediator driving iBRB disruption in DR.

## Discussion

Although glutamate excitotoxicity contributes to retinal neurodegeneration in DR, its role in modulating microglial behavior and vascular pathology remains unclear. Here, we identify a microglial AMPAR subunit composition change (increased GluR1, decreased GluR2) as an upstream sensor of diabetic metabolic stress, which initiates a cascade of calcium-dependent inflammation culminating in iBRB disruption.

Previous studies have already reported the upregulated expression of GluR1 in the retinas of diabetic rats [30, 31]. Consistently, we observed glutamate accumulation and AMPAR subunit changes under hyperglycemic conditions both in vivo and in vitro. These results suggest a close association between hyperglycemia and disrupted glutamatergic signaling in DR. Although diabetes is a highly complex metabolic disorder, and DR progression involves not only hyperglycemia but also other metabolic disturbances (including lipid dysregulation and insulin resistance) as well as disease-stage-related factors, the consistent glutamate accumulation and AMPAR subunit remodeling observed in our type 2 diabetic DR patients and type 1 diabetic DR mice indicate that hyperglycemia plays a central role in abnormal glutamatergic metabolism. Other metabolic and disease-stage factors may act in concert with hyperglycemia, which warrants further exploration.

Mechanistically, GluR2 is a key determinant of AMPAR calcium permeability—GluR2-containing AMPARs are sodium-permeable, while GluR2-lacking receptors gain calcium permeability [32, 33]. Structural and functional studies confirm that loss of GluR2 disrupts canonical assembly, promoting GluR1-homodimeric or GluR1/GluR4-heteromeric CP-AMPARs [34]. These CP-AMPARs adopt distinct assembly patterns mediated by N-terminal domain interactions [35], which correlate with enhanced glutamate responsiveness [34]—a property critically relevant in DR, where HG-induced glutamate accumulation creates a pro-excitotoxic microenvironment. CP-AMPAR expression in microglia is a conserved molecular event [36], and our data establish its pathological engagement in DR. Pharmacological blockade of AMPAR or selective blockade of CP-AMPAR effectively suppressed downstream calcium overload and IL-1β release, positioning CP‑AMPARs as an early metabolic sensor in microglial inflammatory reprogramming.

Calcium overload directly disrupts mitochondrial homeostasis—a process critical for microglial metabolic and inflammatory function [37]. In our study, HG-induced calcium influx in microglia was associated with a loss of mitochondrial membrane potential and dysregulated ATP homeostasis. Elevated extracellular ATP is not a passive metabolic byproduct but a potent danger-associated molecular pattern that amplifies inflammation by engaging purinergic receptors on microglia [38]. This “ATP efflux” converts an intracellular metabolic defect into an extracellular signal that propagates inflammation—establishing a feed-forward loop. P2X7R is a purinergic receptor uniquely positioned to bridge ATP signaling to inflammation, as it is a well-characterized activator of the NLRP3 inflammasome—a multi-protein complex that drives the maturation of pro-inflammatory cytokines [39]. In the present study, we demonstrated that P2X7R activation in the AMPAR‑driven NLRP3/IL‑1β signaling cascade during DR is calcium‑dependent.

IL-1β, an early elevated inflammatory cytokine in DR, contributes critically to retinal inflammation and iBRB damage [40]. Keith et al. found that retinal microglia are a critical source of IL-1β when extracellular ATP levels increase in the retina [41]. In the study of Chemtob et al., retinal IL-1β was primarily secreted by microglia in a rat model of ischemic retinopathy, and this microglia-derived IL-1β induced retinal microvascular injury [42]. Supporting these findings, we observed prominent colocalization of IL-1β with microglia in human diabetic retina, and neutralization of IL-1β alleviated hyperglycemia-induced endothelial dysfunction both in vivo and in vitro. Beyond the iBRB, IL-1β has also been shown to directly disrupts tight junction integrity in retinal pigment epithelium (RPE) cells by downregulating Occludin, which was accompanied by reduced transepithelial resistance and increased permeability [43]. Evidence from both iBRB and RPE studies supports a conserved role of IL-1β in compromising barrier function. These findings, together with our observation in DR mice that microglial activation preceded iBRB impairment and colocalized with focal Occludin loss, support a potential causal link between microglia-derived IL-1β and iBRB breakdown. However, definitive causality remains to be formally established, as IL-1β can also be produced by multiple other retinal cell types besides microglia.

Several limitations of the present study should be noted. First, the AMPAR antagonist PER used in our in vivo study lacks selectivity for microglia or CP‑AMPARs and was not administered via direct intraocular injection. Its protective effects may therefore be mediated by AMPAR inhibition in other AMPAR‑expressing cell types within the retina and CNS, such as neurons and glial cells. Second, our bulk RNA‑seq analysis was performed on whole retinal lysates and therefore cannot resolve microglia‑specific transcriptional profiles; the enriched pathways should thus be interpreted only as a hypothesis‑generating observation. Despite these caveats, our complementary in vitro experiments consistently support a critical role for microglial AMPAR signaling in DR. Specifically, aberrant AMPAR subunit remodeling triggers microglial activation, pathological Ca²⁺ influx, and enhanced IL-1β release, thereby precipitating microglia-induced endothelial dysfunction. Notably, pharmacological AMPAR blockade effectively reverses these deleterious changes. Definitive dissection of cell‑type‑specific roles will require retinal microglia‑specific genetic tools such as GluR1 or GluR2 conditional knockout mice, and combining such strategies with interventions targeting glutamate homeostasis may yield synergistic therapeutic benefits. In addition, the upstream mechanisms underlying glutamate accumulation and AMPAR subunit remodeling in DR remain to be clarified; further investigation into the crosstalk between Müller cell glutamate regulation and microglial AMPAR signaling will help establish a more integrated understanding of retinal metabolic interactions in DR.

## Conclusions

In summary, this study delineates a novel and detailed signaling pathway in DR pathogenesis: hyperglycemia induces retinal glutamate accumulation and CP-AMPARs formation in microglia. This triggers calcium overload, mitochondrial dysfunction, and ATP release, which subsequently activates a specific P2X7R-dependent NLRP3 inflammasome pathway, leading to IL-1β-mediated endothelial dysfunction and iBRB breakdown. Our findings identify microglial CP‑AMPARs as metabolic stress sensors that link glutamatergic signaling to retinal vascular injury, suggesting that targeting the glutamate/AMPAR/P2X7R/IL‑1β axis may represent a rational strategy to maintain iBRB integrity.

## Supplementary Information

Below is the link to the electronic supplementary material.

Supplementary Material 1

## Data Availability

The datasets used and/or analysed during the current study are available from the corresponding author on reasonable request.

## Abbreviations

AMPAR: α-amino-3-hydroxy-5-methyl-4-isoxazolepropionic acid receptors
ANOVA: One-way analysis of variance
BAPTA-AM: 1,2-Bis(2-aminophenoxy) ethane-N, N,N, N-tetraacetic acid tetrakis(acetoxymethyl ester)
BSA: Bovine serum albumin
CM: Conditioned media
CP-AMPAR: Calcium-permeable AMPAR
DMSO: Dimethyl sulfoxide
DR: Diabetic retinopathy
FBS: Fetal bovine serum
GO: Gene Ontology
GSEA: Gene Set Enrichment Analysis
HG: High glucose
HM: High mannitol
iBRB: Inner blood-retina barrier
IF: Immunofluorescence
IL-1β: Interleukin-1β
IL-6: Interleukin-6
LG: Low glucose
NASPM: 1-naphthylacetyl spermine trihydrochloride
NLRP3: NLR family pyrin domain containing 3
PBS: Phosphate-buffered saline
PER: Perampanel
P2X7R: Purinergic Receptor P2X7
P2X4R: Purinergic Receptor P2X4
qPCR: Quantitative real-time polymerase chain reaction
RPE: Retinal pigment epithelium
STZ: Streptozotocin
TNF-α: Tumor Necrosis Factor-α
