# Supplementary material for: AMPAR dysregulation in microglia drives vascular pathology in diabetic retinopathy via the P2X7R/NLRP3/IL-1β pathway
Source: J Transl Med. 2026 Apr 27;24:763. doi: 10.1186/s12967-026-08195-x (PMC13262201; doi:10.1186/s12967-026-08195-x)
Supplement: Supplementary file 1 — Supplementary Material 1 [file 12967_2026_8195_MOESM1_ESM.docx]

**Supplementary data**

**AMPAR Dysregulation in Microglia Drives Vascular Pathology in Diabetic Retinopathy via the P2X7R/NLRP3/IL-1β Pathway**

Lili Zhang, Kaixiang Li, Fengjuan Gao, Jiaojiao Wei, Xin Chen, Gezhi Xu, Yuan Zong, and Ting Zhang

**
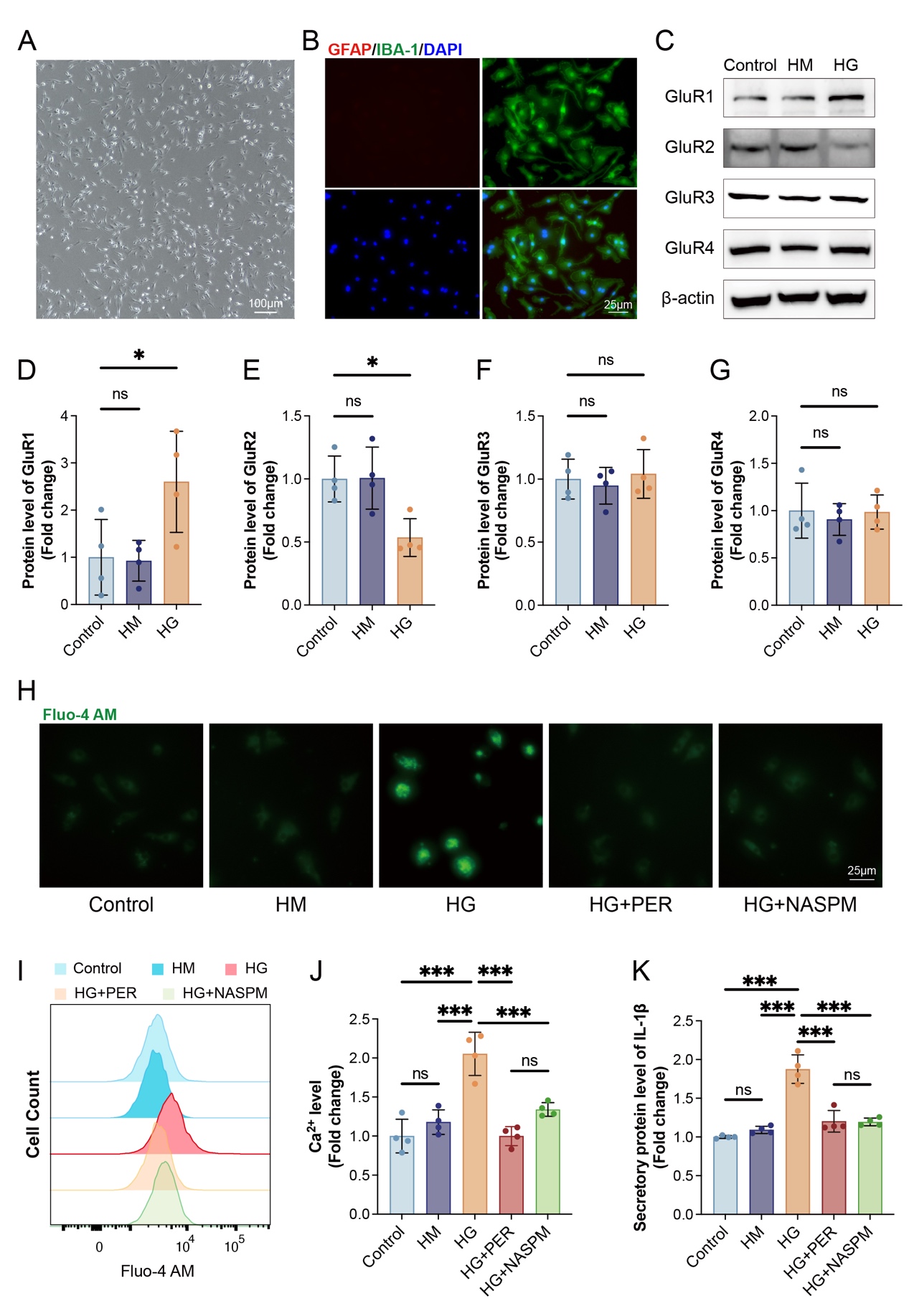
**

**Fig. S1 AMPAR dysregulation induces calcium influx and IL-1β secretion in high glucose-treated primary mouse retinal microglia**

(A) Representative images of in vitro cultured primary mouse retinal microglia. Scale bar: 100 μm. (B) Representative immunofluorescent images of isolated mouse retinal microglia stained with GFAP (Müller cell marker), IBA-1 (microglial cell marker), and DAPI. Scale bar: 25 μm. (C–G) Representative western blot images and quantitative analysis of AMPAR subunits (GluR1–4) in primary mouse retinal microglia. *n* = 4. **P* < 0.05; ns, not significant. One-way ANOVA with Tukey's test. (H) Fluo-4 AM fluorescence imaging detecting intracellular calcium levels in primary mouse retinal microglia. Scale bar: 25 μm. (I–J) Fluo-4 AM flow cytometry analysis of intracellular calcium levels in primary mouse retinal microglia. *n* = 4. ****P* < 0.001; ns, not significant. One-way ANOVA with Tukey's test. (K) ELISA-based quantification of secreted IL-1β levels in primary retinal microglial cell supernatants. *n* = 4. ****P* < 0.001; ns, not significant. One-way ANOVA with Tukey's test. HM: high mannitol; HG: high glucose; PER: perampanel; NASPM: 1-naphthylacetyl spermine trihydrochloride.

**
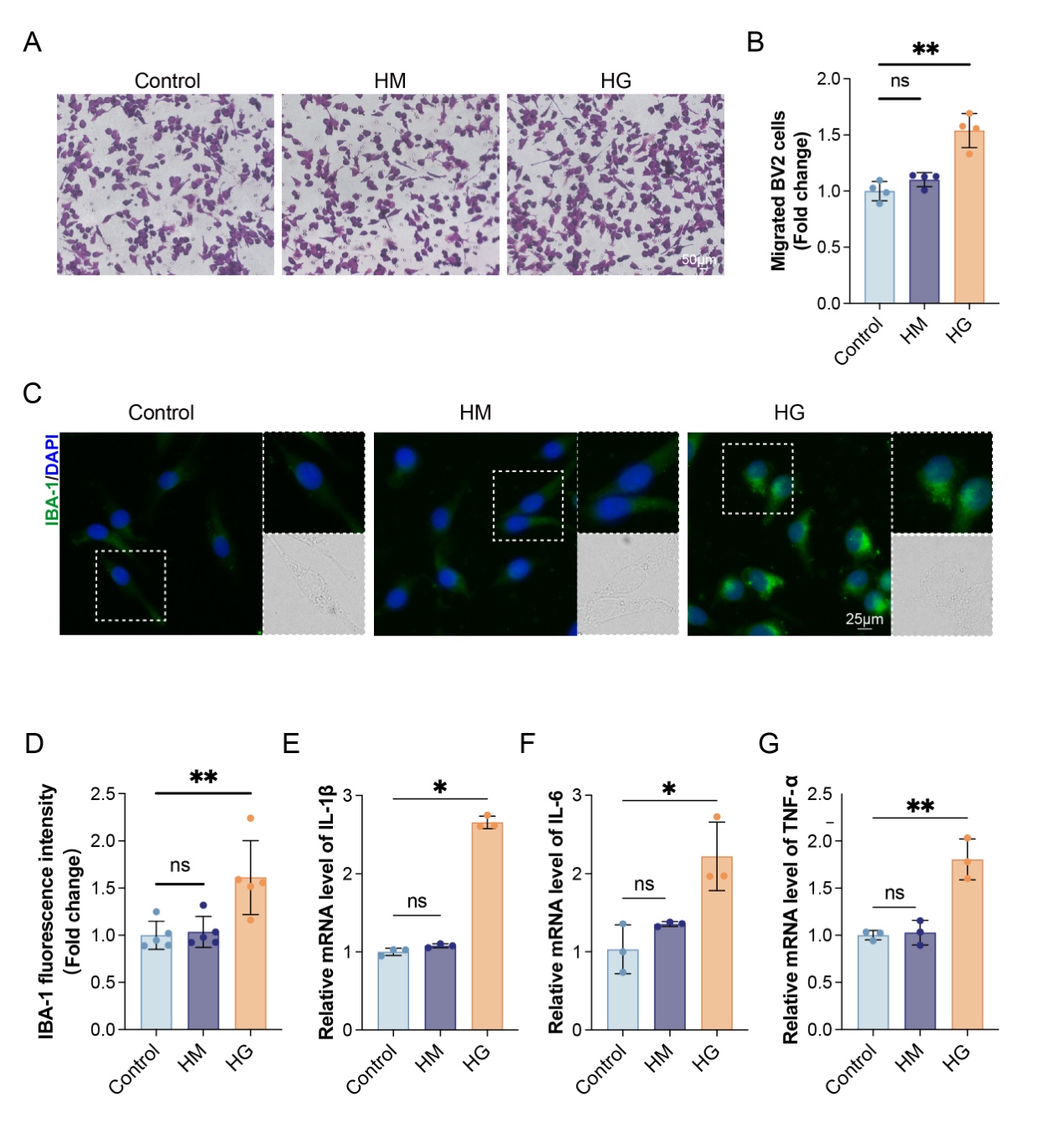
**

**Fig. S2 Hyperosmolarity alone fails to trigger microglia activation**

(A–B) Transwell assay evaluating the migratory capacity of BV2 microglial cells under different treatments, with quantitative analysis. Scale bar: 50 μm. *n* = 4. ***P* < 0.01; ns, not significant. Kruskal–Wallis test with Dunn's test. (C) Representative immunofluorescence staining of IBA-1 and corresponding brightfield images in BV2 cells. Scale bar: 25 μm. (D) Quantitative analysis of IBA-1 staining intensity in BV2 cells. *n* = 5. ***P* < 0.01; ns, not significant. Kruskal–Wallis test with Dunn's test. (E) Relative mRNA expression level of IL-1β in BV2 cells. n = 3. **P* < 0.05; ns, not significant. Kruskal–Wallis test with Dunn's test. (F) Relative mRNA expression level of IL-6 in BV2 cells. *n* = 3. **P* < 0.05; ns, not significant. Kruskal–Wallis test with Dunn's test. (G) Relative mRNA expression level of TNF-α in BV2 cells. *n* = 3. ***P* < 0.01; ns, not significant. One-way ANOVA with Tukey's test. HM: high mannitol; HG: high glucose.


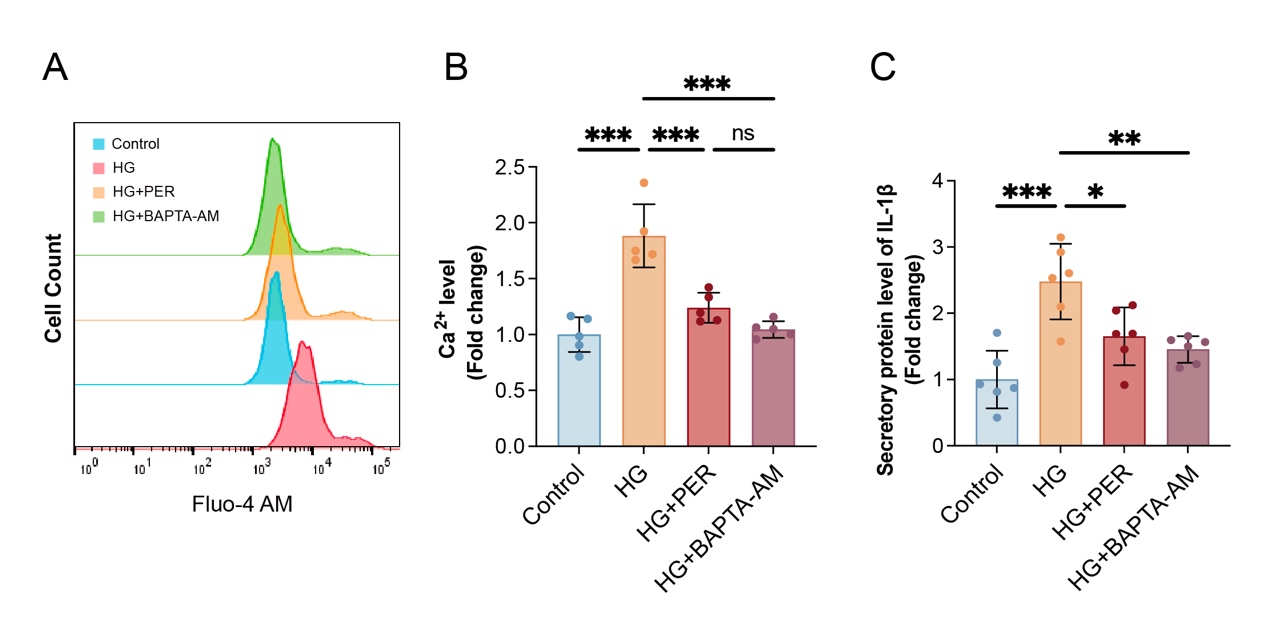


**Fig. S3 Perampanel inhibits high glucose-induced calcium influx and IL-1β secretion in BV2 cells**

(A–B) Fluo-4 AM flow cytometry analysis of intracellular calcium levels in BV2 cells. *n* = 5. ****P* < 0.001; ns, not significant. One-way ANOVA with Tukey's test. (C) ELISA-based quantification of secreted IL-1β levels in BV2 cell supernatants. *n* = 6. **P* < 0.05; ***P* < 0.01; ****P* < 0.001. One-way ANOVA with Tukey's test. HG: high glucose; PER: perampanel; BAPTA-AM: 1,2-Bis(2-aminophenoxy) ethane-N,N,N,N-tetraacetic acid tetrakis(acetoxymethyl ester).


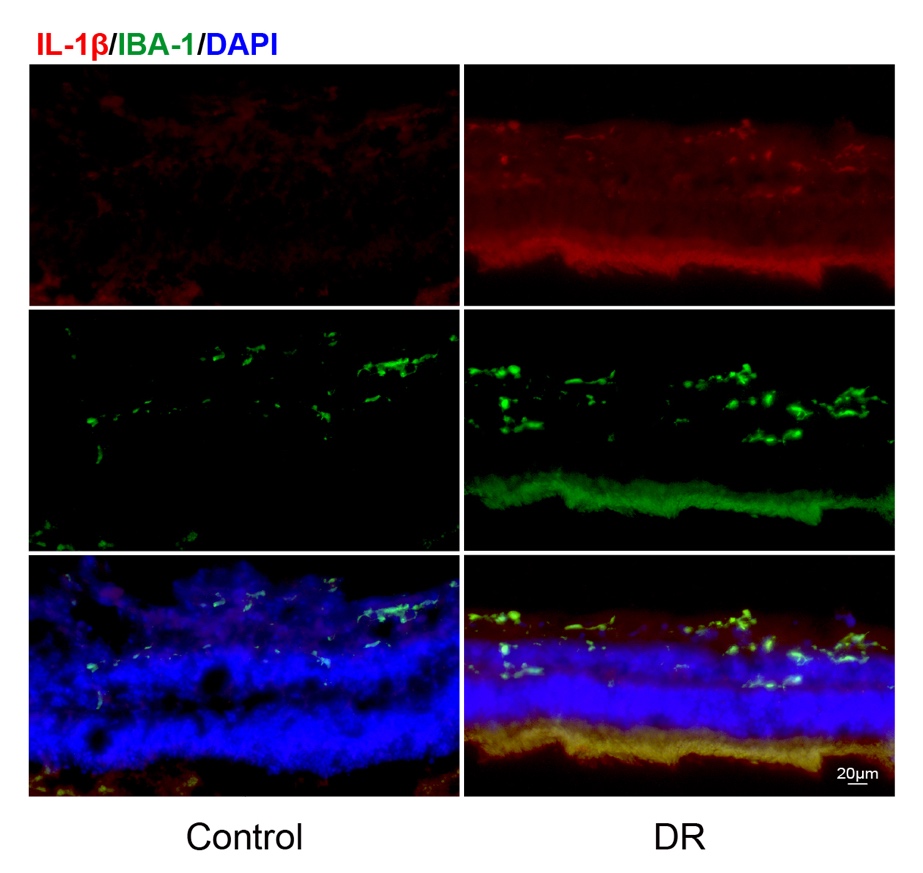


**Fig. S4 IL-1β predominantly co-localized with retinal microglia in DR**

Representative immunofluorescence staining of IBA-1 (microglial marker) and IL-1β in human retinas. Scale bar: 20 μm. DR: diabetic retinopathy.


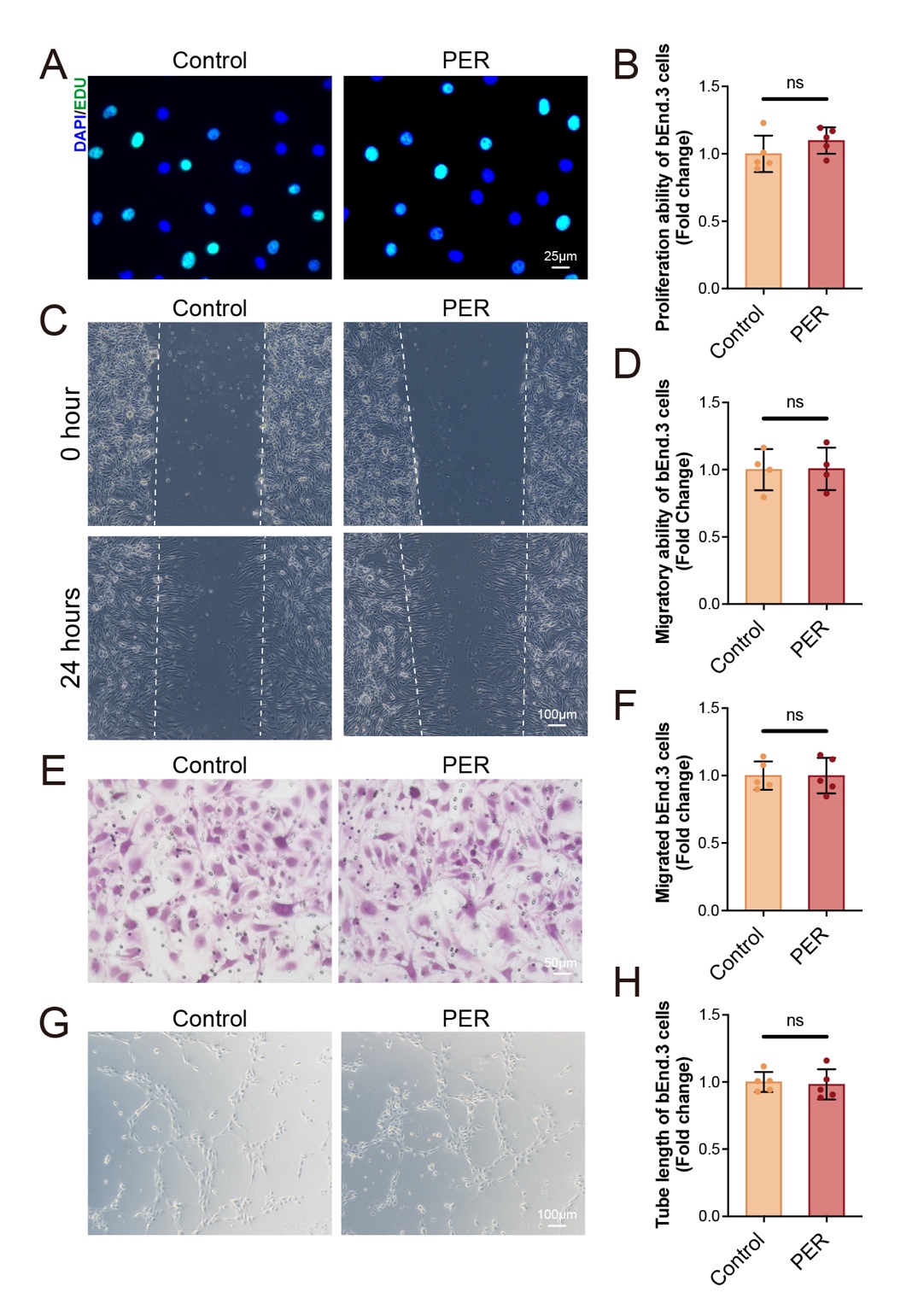


**Fig. S5 PER alone has no direct effects on endothelial cells**

(A–B) EDU assay evaluating the proliferative capacity of bEnd.3 endothelial cells treated with PER or vehicle. Scale bar: 25 μm. *n* = 5. ns, not significant. Unpaired Student *t-*test. (C–D) Wound healing assay assessing the migratory capacity of bEnd.3 cells. Scale bar: 100 μm. *n* = 4. ns, not significant. Unpaired Student *t-*test. (E–F) Transwell assay detecting the migratory capacity of bEnd.3 cells. Scale bar: 50 μm. *n* = 5. ns, not significant. Unpaired Student *t-*test. (G–H) Tube formation assay evaluating the angiogenic capacity of bEnd.3 cells. Scale bar: 100 μm. *n* = 5. ns, not significant. Unpaired Student *t-*test. PER: perampanel.

**
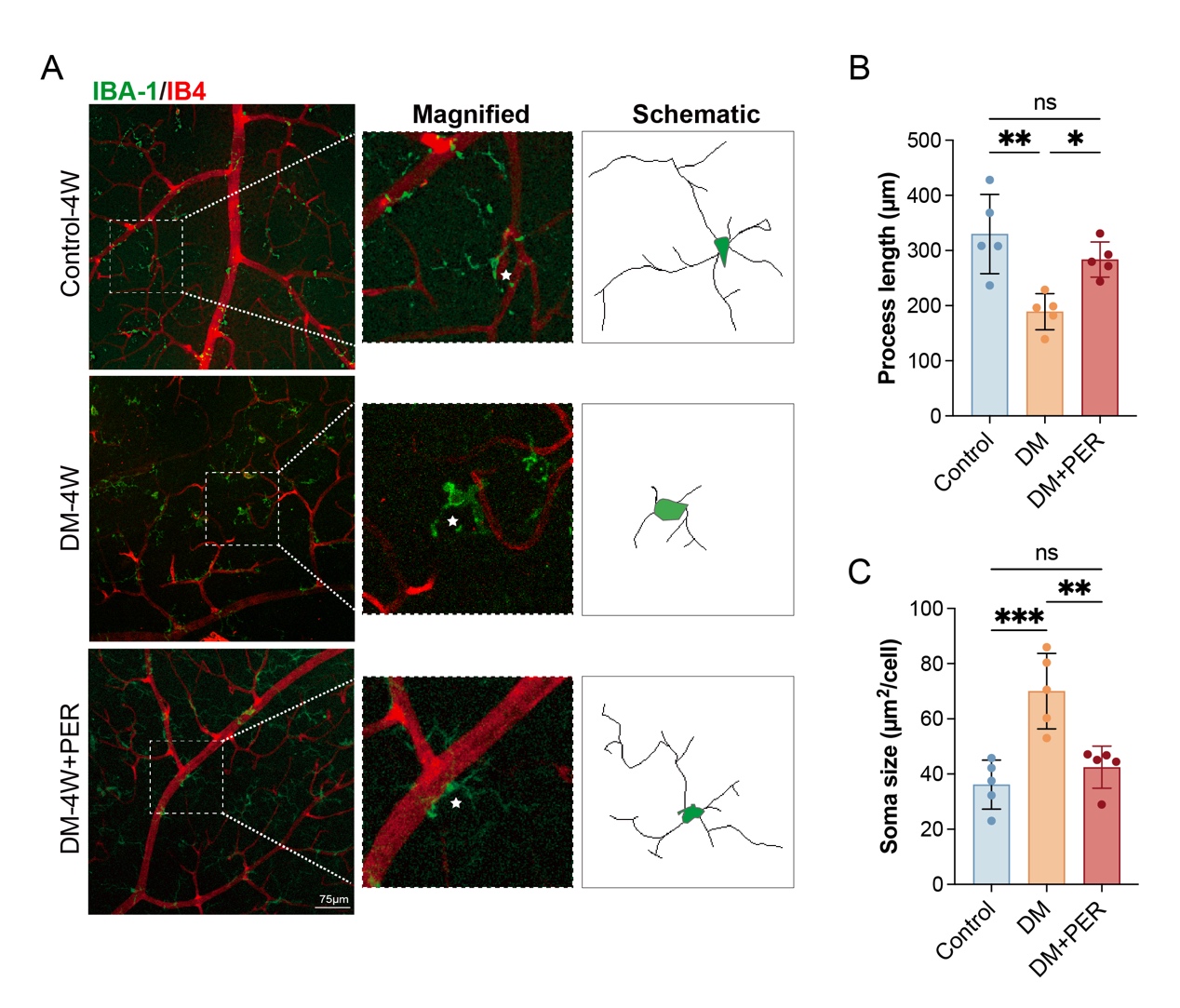
**

**Fig. S6 AMPAR blockade inhibit microglial activation in the early stage of DR**

(A–C) Representative immunofluorescence staining of IBA-1 (microglial marker) and IB4 (vascular marker) in retinas of 4-week control mice, 4-week diabetic mice, and 4-week diabetic mice treated with PER, with quantitative analysis of microglial process length and soma size. Scale bar: 75 μm. *n* = 5. **P* < 0.05; ***P* < 0.01; ****P* < 0.001; ns, not significant. One-way ANOVA with Tukey's test. DM: dabetes mellitus; PER: perampanel.


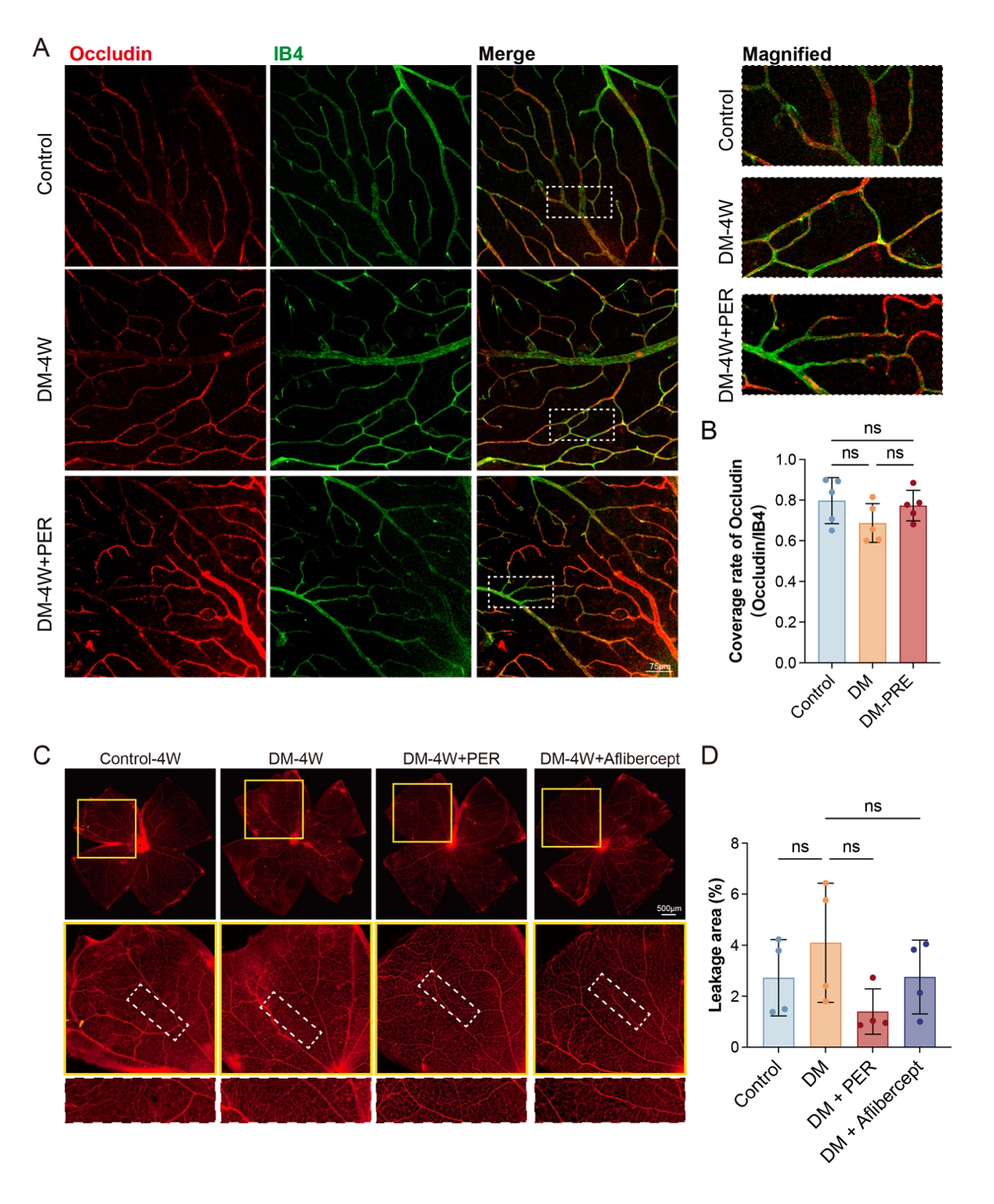


**Fig. S7 No inner brain-retina barrier injury is observed in the early stage of DR**

(A–B) Representative immunofluorescence images and quantitative analysis of Occludin coverage in retinal blood vessels (calculated as Occludin^+^/IB4^+^ ratio) among 4-week control mice, 4-week diabetic mice, and 4-week diabetic mice with PER intervention. Scale bar: 75 μm. *n* = 5. ns, not significant. One-way ANOVA with Tukey's test. (C–D) Representative fluorescence images and quantitative analysis of Evans blue assay, reflecting the amount and distribution of retinal vascular leakage. Scale bar: 500 μm. *n* = 4. ns, not significant. Kruskal–Wallis test with Dunn's test. DM: dabetes mellitus; PER: perampanel.

**Table. S1 Demographic and clinical information of patients with vitreous samples**

| Case | Race | Age (years) | Sex | Eye | Diabetes duration (years) | HbA1c (%) | Diabetes type | HTN (years) | Other systemic disease | Insulin use | Vision acuity (logMAR) | Ophthalmic notes |
| --- | --- | --- | --- | --- | --- | --- | --- | --- | --- | --- | --- | --- |
| 1 | East Asian | 67 | Male | OS | 4 | 8.1 | 2 | No | Coronary heart disease | No | 2.3 | Cataract, VH, FVM, PDR |
| 2 | East Asian | 49 | Female | OD | 15 | 6 | 2 | No | No | Yes | 1.85 | Cataract, VH, PDR, Macular Edema |
| 3 | East Asian | 54 | Male | OD | 5 | 7.2 | 2 | 5 | No | No | 2.3 | Cataract, VH, PDR, ﻿previous PRP history |
| 4 | East Asian | 54 | Female | OS | 22 | 7.9 | 2 | 3 | No | Yes | 2.3 | IOL, VH, PDR |
| 5 | East Asian | 73 | Female | OS | 15 | 7.8 | 2 | 10 | No | Yes | 1.70 | Cataract, VH, Macular Edema, PDR |
| 6 | East Asian | 62 | Female | OS | 2 | 6 | 2 | 1 | No | Yes | 1.40 | Cataract, VH, PDR, |
| 7 | East Asian | 60 | Male | OD | 4 | 7.5 | 2 | No | Renal failure | Yes | 1.85 | IOL, VH, TRD, FVM, PDR |
| 8 | East Asian | 54 | Male | OD | 9 | 7.6 | 2 | 5 | No | No | 0.52 | Cataract, VH, PDR, previous PRP history |
| 9 | East Asian | 52 | Female | OD | 4 | 10.1 | 2 | No | No | Yes | 1.86 | Cataract, TRD, FVM, PDR |
| 10 | East Asian | 42 | Male | OD | 8 | 7.2 | 2 | No | No | No | 2.3 | Cataract, VH, PDR, Macular Edema |
| 11 | East Asian | 69 | Male | OS | No | 5.8 | - | 13 | No | No | 0.82 | Cataract, ERM |
| 12 | East Asian | 74 | Male | OD | No | 5.7 | - | 10 | No | No | 1 | IOL, ERM |
| 13 | East Asian | 67 | Female | OS | No | 5.8 | - | 20 | no | No | 1.10 | Cataract, ERM |
| 14 | East Asian | 68 | Female | OS | No | 6 | - | 5 | Coronary heart disease | No | 0.52 | Cataract, ERM |
| 15 | East Asian | 53 | Male | OS | No | 5.6 | - | No | No | No | 1.40 | Cataract, ERM |
| 16 | East Asian | 66 | Female | OS | No | 5.3 | - | No | No | No | 0.40 | Cataract, ERM |
| 17 | East Asian | 55 | Male | OD | No | 5.3 | - | No | No | No | 1 | Cataract, ERM |
| 18 | East Asian | 60 | Male | OS | No | 5.7 | - | No | No | No | 0.82 | Cataract, Macular Edema, ERM |
| 19 | East Asian | 67 | Male | OS | No | 5.8 | - | 3 | No | No | 1.85 | Cataract, ERM |
| 20 | East Asian | 65 | Female | OS | No | 5.5 | - | No | No | No | 1.85 | Cataract, ERM |

HTN, hypertension; logMAR, logarithm of the minimum angle of resolution; OD, oculus dexter (right eye); OS, oculus sinister (left eye); PRP, pan-retinal photocoagulation; TRD, tractional retinal detachment; VH, vitreous hemorrhage; IOL: intraocular lens; PDR: proliferative diabetic retinopathy; ERM: epiretinal membrane.

**Table. S2 Demographic and clinical information of donor eyes for frozen section preparation**

| Case | Race | Age (years) | Sex | Eye | Diabetes duration (years) | Diabetes type | Systemic disease | Cause of death | Preservation | D-P (hour) | D-R (hour) | Ophthalmic notes |
| --- | --- | --- | --- | --- | --- | --- | --- | --- | --- | --- | --- | --- |
| 21 | East Asian | 74 | Female | OD | 16 | 2 | No | Cerebral infarction | Fixation | 3 | 5 | IOL, DR |
| 22 | East Asian | 67 | Female | OD | 9 | 2 | No | Cardiac arrest | Fixation | 2.5 | 6 | IOL, DR |
| 23 | East Asian | 50 | Male | OS | 10 | 2 | Diabetes nephropathy, Hypertension | Renal failure | Fixation | 2 | 5 | Cataract, DR |
| 24 | East Asian | 79 | Male | OS | - | - | Pulmonary embolism | Type 2 respiratory failure | Fixation | 2 | 6 | IOL |
| 25 | East Asian | 73 | Femae | OD | - | - | Colon cancer | Colon cancer | Fixation | 2 | 4 | IOL |
| 26 | East Asian | 56 | Male | OS | - | - | Hypertension | Cerebral hemorrhage | Fixation | 3 | 5.5 | Cataract |
| 27 | East Asian | 50 | Male | OD | 8 | 2 | Coronary heart disease | Cardiac arrest | Fixation | 2.5 | 5.5 | Cataract, DR |
| 28 | East Asian | 62 | Female | OS | 21 | 2 | Hypertension | Sudden death | Fixation | 2 | 5 | IOL, DR |
| 29 | East Asian | 61 | Male | OD | 13 | 2 | Stroke | Cerebral infarction | Fixation | 2 | 5.5 | IOL, DR |
| 30 | East Asian | 54 | Male | OD | - | - | No | Cerebral infarction | Fixation | 3 | 6 | Cataract |
| 31 | East Asian | 65 | Male | OD | - | - | No | Pneumonia | Fixation | 2 | 4.5 | IOL |
| 32 | East Asian | 68 | Female | OD | - | - | Chronic kidney disease | Renal failure | Fixation | 2 | 5 | Cataract |

OD, oculus dexter (right eye); OS, oculus sinister (left eye); DR: diabetic retinopathy; D-P: Time interval from death to preservation; D-R: Time interval from death to receipt.

**Table. S3 Demographic and clinical information of donor eyes for western blot**

| Case | Race | Age (years) | Sex | Eye | Diabetes duration (years) | Diabetes type | Systemic disease | Cause of death | Preservation | D-P (hour) | D-R (hour) | Ophthalmic notes |
| --- | --- | --- | --- | --- | --- | --- | --- | --- | --- | --- | --- | --- |
| 21 | East Asian | 74 | Female | OS | 16 | 2 | No | Cerebral infarction | -80 °C | 3 | 5 | IOL, DR |
| 22 | East Asian | 67 | Female | OS | 9 | 2 | No | Cardiac arrest | -80 °C | 2.5 | 6 | IOL, DR |
| 24 | East Asian | 79 | Male | OD | - | - | Pulmonary embolism | Type 2 respiratory failure | -80 °C | 2 | 6 | IOL |
| 25 | East Asian | 73 | Femae | OS | - | - | Colon cancer | Colon cancer | -80 °C | 2 | 4 | IOL |
| 26 | East Asian | 56 | Male | OD | - | - | Hypertension | Cerebral hemorrhage | -80 °C | 3 | 5.5 | Cataract |
| 28 | East Asian | 62 | Female | OD | 21 | 2 | Hypertension | Sudden death | -80 °C | 2 | 5 | IOL, DR |
| 30 | East Asian | 54 | Male | OS | - | - | No | Cerebral infarction | -80 °C | 3 | 6 | Cataract |
| 31 | East Asian | 65 | Male | OS | - | - | No | Pneumonia | -80 °C | 2 | 4.5 | IOL |
| 32 | East Asian | 68 | Female | OS | - | - | Chronic kidney disease | Renal failure | -80 °C | 2 | 5 | Cataract |
| 33 | East Asian | 62 | Male | OD | 12 | 2 | Diabetic nephropathy | Renal failure | -80 °C | 3.5 | 6 | Cataract, DR |
| 34 | East Asian | 52 | Female | OS | 7 | 2 | Hypertension | Pneumonia | -80 °C | 3 | 5.5 | Cataract, DR |
| 35 | East Asian | 72 | Male | OS | 25 | 2 | Hypertension | Cerebral infarction | -80 °C | 4 | 7 | IOL, DR |

OD, oculus dexter (right eye); OS, oculus sinister (left eye); DR: diabetic retinopathy; D-P: Time interval from death to preservation; D-R: Time interval from death to receipt.

| **Table. S4 Antibody Information** | | | | |
| --- | --- | --- | --- | --- |
| **Antibody Name** | **Catalog number** | **Company** | **Dilution Ratio** | **Application** |
| Isolectin B4 antibody (IB4) | Cas# I21411 | Invitrogen | 1:100 | IF |
| Occludin antibody | Cas# 740006M | Invitrogen | 1:100 | IF |
| IBA-1 antibody | ab178846 | Abcam | 1:100 | IF |
| GluR1 antibody | Cas# A11643 | Abclonal | 1:100; 1:1000 | IF; WB |
| P2X7R antibody | ab307718 | Abcam | 1:100; 1:1000 | IF; WB |
| P2X4R antibody | ab134559 | Abcam | 1:100 | IF |
| GluR2 antibody | ab206293 | Abcam | 1:1000 | WB |
| NLRP3 antibody | ab283819 | Abcam | 1:1000 | WB |
| Caspase-1 antibody | Cas# ET1608-69 | Huabio | 1:1000 | WB |
| GFAP antibody | Cat No. 60190-1-Ig | Proteintech | 1:100 | IF |
| IL-1β antibody | ab177475 | Abcam | 1:100 | IF |
| Goat anti-Mouse IgG (H+L), Alexa Fluor Plus 488 | Cat# A32723 | Invitrogen | 1:500 | IF |
| Goat anti-Rabbit IgG (H+L), Alexa Fluor Plus 488 | Cat# A32731 | Invitrogen | 1:500 | IF |
| Goat anti-Rabbit IgG (H+L), Alexa Fluor^TM^ Plus 555 | Cat# A32732 | Invitrogen | 1:500 | IF |
| Goat anti-Mouse IgG (H+L), Alexa Fluor^TM^ Plus 555 | Cat# A32727 | Invitrogen | 1:500 | IF |
| IL-1β neutralizing antibody | MAB4012 | R & D | 0.5 ng/eye | I.V. |
| isotype control IgG | 14-4888-81 | Invitrogen | 0.5 ng/eye | I.V. |

IF: Immunofluorescence; WB: western blotting; I.V.: intravitreal injection.

| **Table. S5 Primer information** | |
| --- | --- |
| **Primer name** | **Primer sequence (5’-3’)** |
| IL-1β | F: TGGACCTTCCAGGATGAGGACA  R: GTTCATCTCGGAGCCTGTAGTG |
| TNF-α | F: AGTGACAAGCCTGTAGCCCA  R: GTCTGGTAGGAGACGGCGAT |
| IL-6 | F: TACCACTTCACAAGTCGGAGGC  R: CTGCAAGTGCATCATCGTTGTTC |
| β-Actin | F1: GGCTGTATTCCCCTCCATCG  R1: CCAGTTGGTAACAATGCCATGT |
| F: forward primer; R: reverse primer. | |
